# Dysregulation of Hippocampal Autophagic Machinery in the Activity‐Based Anorexia Rat Model

**DOI:** 10.1111/jnc.70549

**Published:** 2026-09-10

**Authors:** Beatrice Rizzi, Giorgia Targa, Sofia Taddini, Susanna Parolaro, Letizia Rapini, Fabio Fumagalli, Lucia Caffino, Francesca Mottarlini

**Affiliations:** ^1^ Department of Pharmacological and Biomolecular Sciences ‘Rodolfo Paoletti’ Università Degli Studi di Milano Milano Italy

**Keywords:** adolescence, autophagy, dorsal hippocampus, eating disorder

## Abstract

In Anorexia Nervosa (AN), the combination of self‐induced starvation and hyperactivity may initially recruit the autophagic machinery to sustain the function of energy‐demanding brain regions; however, its dysregulation may ultimately contribute to impaired brain function. We therefore investigated the autophagic machinery in the dorsal hippocampus (dHip), a central hub for energy status modulation and for cognitive processing, in the activity‐based anorexia (ABA) rat model, the gold standard in the field. To analyze autophagic markers in the dHip, adolescent female Sprague–Dawley rats were exposed to 2 h/day of food access + free wheel access (ABA induction) and were sacrificed when they reached the maximum body weight loss allowed concomitantly with an increase in the running activity (acute phase) or following a 7‐day recovery period. Our results show that ABA induction persistently disrupts the regulation of the autophagic machinery. In the acute phase, the ABA condition increases the phosphorylation of mTOR/ULK1 while promoting TFEB‐associated mechanisms, enhancing the expression of autophagic markers such as *Becn1*, *Ctsb* genes, Beclin‐1, and LC3‐II proteins. After weight recovery, TFEB‐induced activation of *Becn1*, *Ctsb*, Beclin‐1, and LC3‐II remains elevated. In addition, the enhanced expression of autophagic markers is accompanied by an increase in p62 and Caspase3, pro‐apoptotic signals. Our findings reveal a sustained activation of autophagy across different stages of the ABA protocol. Rather than functioning exclusively as a survival mechanism, this process could evolve into a driver of aberrant behaviors typical of individuals with AN, such as dieting behaviors and hippocampal‐dependent deficits.

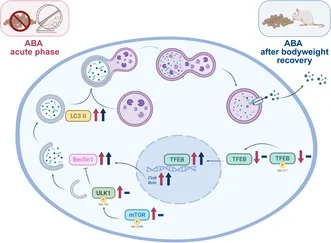

## Graphical abstract

Dysregulation of autophagy pathway in the dorsal hippocampus of Activity‐Based Anorexia (ABA) female rats during the acute phase and after seven days of bodyweight recovery. ABA rats at both acute phase and after seven days of bodyweight recovery show upregulation of core autophagic markers (Beclin1, LC3‐II) and increased nuclear translocation of TFEB, driving the transcription of autophagy‐related genes (*Ctsb: Cathepsin B*, *Bcln: Beclin‐1*). Red arrows indicate expression and phosphorylation changes during the ABA acute phase, while blue arrows/lines represent changes observed after seven days of bodyweight recovery. The direction of the arrows indicates either increase, decrease, or unchanged expression of the relative target.

**Abbreviations**
ABA: activity‐based anorexia
AN: anorexia nervosa
*Becn1*
: *Beclin‐1*
CTRL: control group
*Ctsb*
: *Cathepsin B*
dHip: dorsal hippocampus
EXE: exercise group
FR: food restricted group
LC3‐II: microtubule‐associated protein 1A/1B‐light chain 3
mTOR: mammalian target of rapamycin
PND: postnatal day
TFEB: transcription factor EB
ULK1: Unc‐51‐like autophagy activating kinase 1

## 1 Introduction

Autophagy is a catabolic process that maintains cellular homeostasis by regulating energy sources and preserving cellular functions by degrading and recycling cytoplasmic components of the cell (Nikoletopoulou and Tavernarakis 2018). Changes in nutrient availability engage autophagic processes: in fact, a condition of starvation increases the autophagic flux as an alternative energy source to sustain cell integrity and functions. Interestingly, it has been demonstrated that fasting regulates autophagy in a brain region‐dependent manner. Food deprivation in mice inhibits autophagy in the forebrain while increasing it in the hypothalamus (Glatigny et al. 2019), suggesting that these fine‐tuned mechanisms might contribute to alter neuronal functionality and consequently behavioral outcomes.

From a mechanistic point of view, the autophagic process is primarily regulated by mammalian target of rapamycin (mTOR), a nutrient‐sensing kinase, which inhibits autophagy via phosphorylation of Unc‐51‐like autophagy activating kinase 1 (ULK1) under nutrient‐rich conditions (Hosokawa et al. 2009; Howell and Manning 2011; Kim et al. 2011). mTOR, when activated, also represses the activity of transcription factor EB (TFEB), a key transcription factor for lysosomal and autophagy‐related genes, by preventing its nuclear translocation through phosphorylation. During starvation, mTOR inhibition allows TFEB activation and transcriptional upregulation of the autophagy–lysosome pathway (Napolitano and Ballabio 2016; Settembre et al. 2012). While autophagy primarily maintains cellular homeostasis, it also mediates non‐apoptotic cell death (Glick et al. 2010; Jung et al. 2020; S. Liu et al. 2023) and supports hippocampal‐dependent cognitive functions by regulating neuronal homeostasis and plasticity (Glatigny et al. 2019; Nikoletopoulou and Tavernarakis 2018). Of note, dysregulation of autophagic mechanisms in the central nervous system during development as well as in response to stressful conditions has been associated with different pathological conditions, including neurodevelopmental, neurodegenerative and psychiatric disorders (Griffey and Yamamoto 2022).

In this framework, anorexia nervosa (AN), an eating disorder characterized by a lack of nutrient availability and aberrant energy expenditure (Misra and Klibanski 2014; Pirke et al. 1991), could potentially lead to a dysregulation of the autophagic machinery in brain regions showing structural and functional abnormalities in individuals diagnosed with AN. However, at present, there is no direct evidence supporting this link.

Subjects that suffer from AN exert overcontrol over food intake by self‐imposing unhealthy dietary restrictions that, combined with hyperactive behaviors, lead to the desired body weight loss (American Psychiatric Association 2022). The low food intake fails to sustain the high energy requirements, resulting in metabolic, gastrointestinal, and cardiovascular dysfunctions and low bone mineral density. Moreover, reduction in brain volumes in underweight individuals with AN has been observed in highly energy demanding cortical and subcortical brain regions, areas also correlated with increased susceptibility to AN phenotype (Bahnsen et al. 2022). It has been proposed that this effect could be ascribed to a reduced number of glial and neuronal cells driven by neuroplastic and mitochondrial dysfunctions, as observed in the postmortem brain of subjects with AN and in multimodal studies of neuroimaging and biomarkers in the blood (Doose et al. 2023; Gaiaschi et al. 2024; Seitz et al. 2021). A similar reduction in brain volume, in astrocyte, and in synaptic and metabolic markers has been observed in the brain of Activity‐Based Anorexia (ABA) rats, the gold standard rodent model of AN consisting of the combination of food restriction and hyperactivity (Frintrop et al. 2019; Rousseau et al. 2025).

This translational parallelism has been particularly explored in the hippocampus, a central hub for cognitive processing, which has been increasingly recognized as a crucial target in the modulation of energy status in eating behaviors (Kanoski and Grill 2017; Malá et al. 2015). Reduced hippocampal volumes have been positively correlated with body mass index in individuals with AN (Collantoni et al. 2021; Myrvang et al. 2018) and decreased intra‐connectivity in the hippocampal network in AN might contribute to impaired cognitive flexibility (Myrvang et al. 2021; Olivo et al. 2019). Hippocampal‐dependent functional and molecular alterations in AN have been further corroborated in ABA rats showing memory impairment, significant reorganization of hippocampal synapses (Bilash et al. 2021; Chowdhury, Ríos, et al. 2014), which persisted even after bodyweight recovery (Mottarlini et al. 2024), altered mitochondrial functioning (Bhasin et al. 2023) and neuroinflammatory and neuroplastic markers (Spero et al. 2024). Such a reduced hippocampal volume and an impaired structural and functional state induced by the combination of food restriction and hyperactivity may underlie unbalanced mechanisms responsible for the proper functioning of cells, such as autophagy and cell death. While enhanced activation of autophagy in the hypothalamus of female mice exposed to the ABA model has already been demonstrated as a short‐term protective strategy to provide energy under nutrient‐deprived conditions (Nobis et al. 2018), its role in the dorsal hippocampus (dHip) remains unknown. This subregion, involved in spatial memory, is found impaired in subjects diagnosed with AN (Stedal et al. 2022; Wang et al. 2026) as well as in ABA rats and it is known to contribute to the maintenance of the AN phenotype in ABA rodents (Chowdhury, Ríos, et al. 2014; Mottarlini et al. 2024; Targa et al. 2024).

Thus, in this work we investigated the impact of the AN phenotype during adolescence on the autophagic machinery in the dHip of female ABA rats at the achievement of the maximum allowed bodyweight loss and hyperactivity (éacute phase, post‐natal day (PND) 42) and after a week of body weight recovery (PND49).

## 2 Materials and Methods

### 2.1 Animals and Housing

The experimental procedures were performed on 40 adolescent female Sprague–Dawley rats (Charles River, Calco, Italy, RRID: RGD_734476) at the Department of Pharmacological and Biomolecular Sciences “Rodolfo Paoletti”, University of Milan. Given the higher prevalence of AN among females (American Psychiatric Association 2022), only female rats were used in the present study. All animal‐related protocols adhered to ethical standards set out in the subsequent laws and policies governing the care and use of laboratory animals: Italian Governing Law (D.lgs 26/2014; Authorization #861_2023_PR issued by Italian Ministry of Health); the NIH Guide for the Care and Use of Laboratory Animals (2011 edition) and EU directives and guidelines (EEC Council Directive 2010/63/UE). Every effort was made to minimize distress and limit the number of animals used. To uphold ethical considerations, ABA rats were prevented from losing more than 25% of their initial bodyweight. The study adheres to the ARRIVE guidelines for reporting animal research (Percie Du Sert et al. 2020).

Upon arrival, rats were housed under controlled environmental conditions, with a temperature of 21°C ± 1°C and humidity levels maintained between 50% and 60%. A reversed 12‐h light/dark cycle was set (lights on at 10:30 pm; lights off at 10:30 am). Animals had access to standard rat chow (ssniff Spezialdiäten GmbH, Soest, Germany) and tap water ad libitum. To prevent potential “litter effects,” a maximum of two female siblings were selected from each litter (Chapman and Stern 1978). No randomization methods were used to allocate subjects in the study; the animals were arbitrarily divided by chance into the experimental groups.

### 2.2 Experimental Design

Forty adolescent female rats arrived on post‐natal day (PND) 28 and were initially housed in groups of 3 within standard home cages, as described in Mottarlini et al. (2022). They remained undisturbed to acclimate to the reversed light/dark cycle until PND31, at which point daily weight measurements commenced. At PND35, animals were individually housed, with cages arranged nearly to provide visual, auditory, and olfactory contact. Animals were arbitrarily divided into four groups, ensuring that siblings, if present, were assigned to different experimental conditions: control (CTRL) group, housed in standard home cages with food ad libitum (*n* = 10); food‐restricted (FR) group, housed in standard home cages with food limited for 2 h/day (*n* = 10); exercise (EXE) group, allowed to freely access a mechanical wheel (activity wheel BIO‐ACTIVW—R cage, Bioseb, France) to experience voluntary running activity with food ad libitum (*n* = 10); activity‐based anorexia (ABA) group, allowed to freely access a mechanical wheel to experience voluntary running activity with food limited for 2 h/day (*n* = 10).

After 3 days of acclimatization to the new environment, at PND38, the food restriction started for FR and ABA rats which were given only 2 h/day access to an unlimited amount of food from 10.30 to 12.30 a.m. Wheels of EXE and ABA rats were blocked during these 2 h. At PND42, ABA rats reached the anorexic phenotype, defined by a bodyweight loss equivalent to 25% compared to their expected physiological bodyweight alongside a sharp increment in wheel activity (Carrera et al. 2014). Half of the rodents (*n* = 5/experimental group, for a total of *n* = 20) were sacrificed by decapitation at PND42 (acute phase), while the remaining animals (*n* = 5/experimental group, for a total of *n* = 20) were transferred into standard housing cages with food ad libitum to allow bodyweight recovery until sacrifice on PND49.

The dHip brain region was freshly dissected from the whole brain −2.16 mm to bregma −6.12 mm according to the Paxinos and Watson Rat Brain Atlas (Paxinos and Watson 2014), rapidly frozen on dry ice and stored at −80°C. These brain areas were collected in a previous study; in fact, daily measurements of body weight, food consumption, and wheel‐running behavior for each animal are reported in a previously published research (Mottarlini et al. 2022).

### 2.3 mRNA Isolation and Real‐Time PCR Analysis

Total RNA was isolated from dHip homogenate in the TissueLyser (Qiagen, RRID:SCR_020428) (30 Hz, 30 s) by single step guanidinium isothiocyanate/phenol extraction via PureZol RNA isolation reagent (Bio‐Rad Laboratories, Segrate, Milan, Italy, Cat#7326890) and quantified by spectrophotometric analysis. Samples were processed for real‐time reverse transcription polymerase chain reaction (real‐time RT‐PCR, Bio‐Rad Laboratories, RRID:SCR_018057) to assess mRNA levels as previously reported (Mottarlini et al. 2022).

RT‐PCR analysis was performed to assess *Cathepsin B* (*Ctsb*) and *Beclin‐1* (*Becn1*) mRNA levels. Data were analyzed using the comparative threshold cycle (DDCt), with *36B4* as the internal standard. Primers and probes for *Ctsb* and *Becn1* were purchased from Applied Biosystem, Foster City, California (*Ctsb*: ID Rn00575030_m1 and *Becn1*: ID Rn00586976_m1). Primers and probes for *36B4* were purchased from Eurofins MWG‐Operon. Its sequence is shown below:

*36B4*: forward primer 5′‐TCAGTGCCTCACTCCATCAT‐3′, reverse primer 5′‐AGGAAGGCCTTGACCTTTTC‐3′, probe 5′‐TGGATACAAAAGGGTCCTGG‐3′.

### 2.4 Protein Isolation and Western Blot Analysis

Proteins extraction from dHip and Western blots were performed as previously reported with minor modifications (Targa et al. 2024). Briefly, tissues from dHip were homogenized in a glass–glass potter using cold sucrose buffer pH 7.4 containing 1 mM HEPES (Sigma‐Aldrich, Cat#H706), 0.1 mM PMSF (Merck, Cat# 52332), and 0.1 mM EGTA (Sigma‐Aldrich, Cat# E4378) in the presence of a commercial cocktail of protease (cOmplete Protease Inhibitor Cocktail, Roche, Monza, Italy, Cat# 1169749800) and phosphatase (Sigma‐Aldrich, Milan, Italy, Cat#P5726). An aliquot of each sample was stored as homogenate fraction. The remaining homogenate was centrifuged obtaining a pellet (P1) corresponding to the nuclear fraction and a supernatant (S1). The resulting P1 was resuspended in a buffer solution (20 mM HEPES, 0.1 mM DDT, 0.1 mM EGTA pH 7.4, 1× Complete and 1× phosphatase inhibitors cocktail), while the S1 was centrifuged again. The resulting supernatant corresponds to the clarified fraction of cytosolic proteins (S2). Eventually, all the fractions were stored at −20°C until the molecular analysis.

Equal amount of proteins (10 μg) were run on a sodium dodecyl sulphate 8% (P1 and S2 extracts) or 14% (homogenate extracts) polyacrylamide gel under reducing conditions and then transferred onto nitrocellulose membranes (GE Healthcare). Blots were blocked for 1 h at room temperature with I‐Block solution (Life Technologies Italia, Italy) in TBS 0.1% Tween‐20 buffer and washed with TBS 0.1% Tween‐20 solution. Proteins of interest were detected by probing with the specific primary antibody followed by HRP‐conjugated secondary antibodies (Anti‐rabbit IgG, HRP‐linked Antibody, Cell Signaling Technology Cat#7074, RRID:AB_2099233**;** Anti‐mouse HRP‐linked Antibody polyclonal antibody, Sigma‐Aldrich Cat#A4416 RRID:AB_258167). The entire nitrocellulose blot (Bio‐rad Laboratories, Cat#1620115) was cut near the positions corresponding to the expected molecular weight of target proteins, as indicated by their specific molecular weight and the antibody datasheets. The same procedure was performed to assess successful subcellular fractionation, by loading 10 μg of homogenate, S2 and P1 extracts from the same control samples on a 14% polyacrylamide gel and probing the membranes with anti‐GAPDH (1:3000, Cell Signaling Technology Cat# 97166, RRID:AB_2756824) and anti‐H3 (1:1000, Cell Signaling Technology Cat# 4499, RRID:AB_10544537) as markers of cytosolic and nuclear fractions, respectively (Figure S7).

Primary antibodies conditions were the following: anti‐pmTOR_S2448_ (1:1000, Cell Signaling Technology Cat# 5536, RRID:AB_10691552), anti‐mTOR (1:1000, Cell Signaling Technology Cat# 2983, RRID:AB_2105622), anti‐pULK1_S757_ (1:1000, Cell Signaling Technology Cat# 14202, RRID:AB_2665508), anti‐ULK1 (1:1000, Cell Signaling Technology Cat# 8054, RRID:AB_11178668), anti‐pTFEB_S211_ (1:1000, Invitrogen, Cat# PA5‐114662, RRID:AB_2899298), anti‐TFEB (1:1000, Invitrogen, Cat# PA5‐96632, RRID:AB_2808434), anti‐Beclin‐1 (1:1000, Cell Signaling Technology Cat# 3495, RRID:AB_1903911), anti‐LC3‐II (1:1000, Invitrogen, Cat#: PA1‐46286, RRID:AB_2234770), anti‐p62 (1:1000, Sigma‐Aldrich Cat# P0067, RRID:AB_1841064), anti‐Caspase3 (1:1000, Cell Signaling Technology Cat# 9662, RRID:AB_331439), anti‐CRMP2 (1:2000, Cell Signaling Technology Cat# 9393, RRID:AB_2094339). Results were standardized to β‐actin control protein (1:7500, Sigma Aldrich, cod: A5441, RRID: AB_476744) detected at 43 kDa.

**FIGURE 1:**
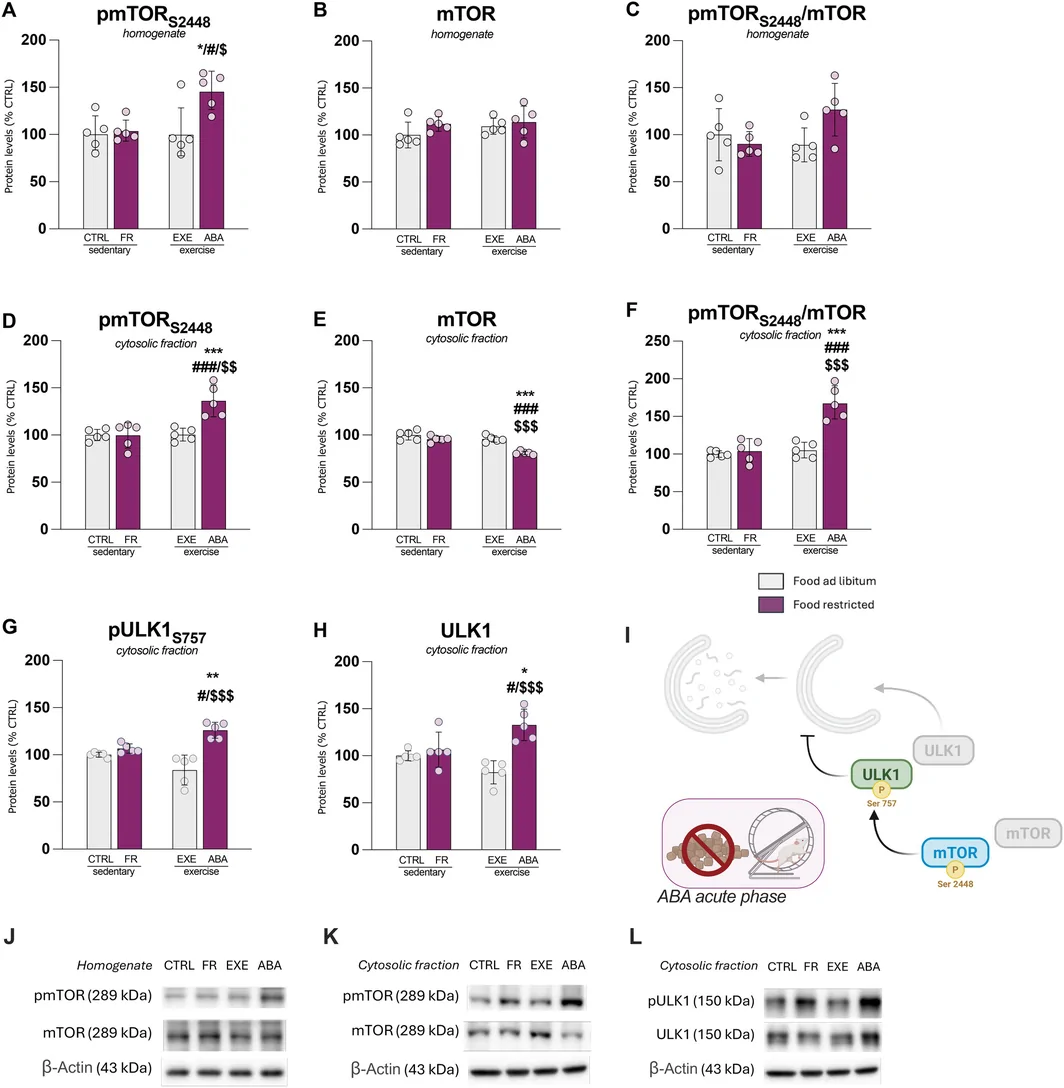
The induction of the ABA phenotype alters mTOR and ULK1 protein levels in the dorsal hippocampus at the acute phase. Panels (A), (B), and (C) show pmTOR_Ser2448,_ mTOR protein levels and the relative ratio pmTOR_Ser2448_/mTOR, respectively, in the whole homogenate of CTRL, FR, EXE, and ABA rats, while panels (D), (E), and (F) display pmTOR_Ser2448_, mTOR protein levels and the relative ratio pmTOR_Ser2448_/mTOR, respectively, in the cytosolic fraction. Panels (G) and (H) show protein pULK1_Ser757_ and ULK1 protein levels in the cytosolic fraction, respectively. Representative immunoblots for each protein are shown in panels (J–L). Panel (I) provides a graphical representation of the autophagy molecular cascade in the ABA group, highlighting the targets described in the adjacent panels: colored elements indicate pathway activation; gray elements represent inactive components. In ABA rats, phosphorylation of mTOR at S2448 suppresses autophagy by phosphorylating ULK at S757. Data are expressed as percentage (%) of CTRL and represent the mean ± SD (B–E, G, H) or the geometric mean ± geometric SD (A, F) of each group. Two‐way ANOVA followed by Tukey's multiple comparison test *p < 0.05, **p < 0.01, ***p < 0.001 versus CTRL; ^#^ *p* < 0.05, ^###^ *p* < 0.001 versus FR; ^$^ *p* < 0.05, ^$$^ *p* < 0.01, ^$$$^ *p* < 0.001 versus EXE. ABA, activity‐based anorexia (*n* = 5); CTRL, control (*n* = 5); EXE, exercise (*n* = 5); FR, food restricted (*n* = 5).

**FIGURE 2:**
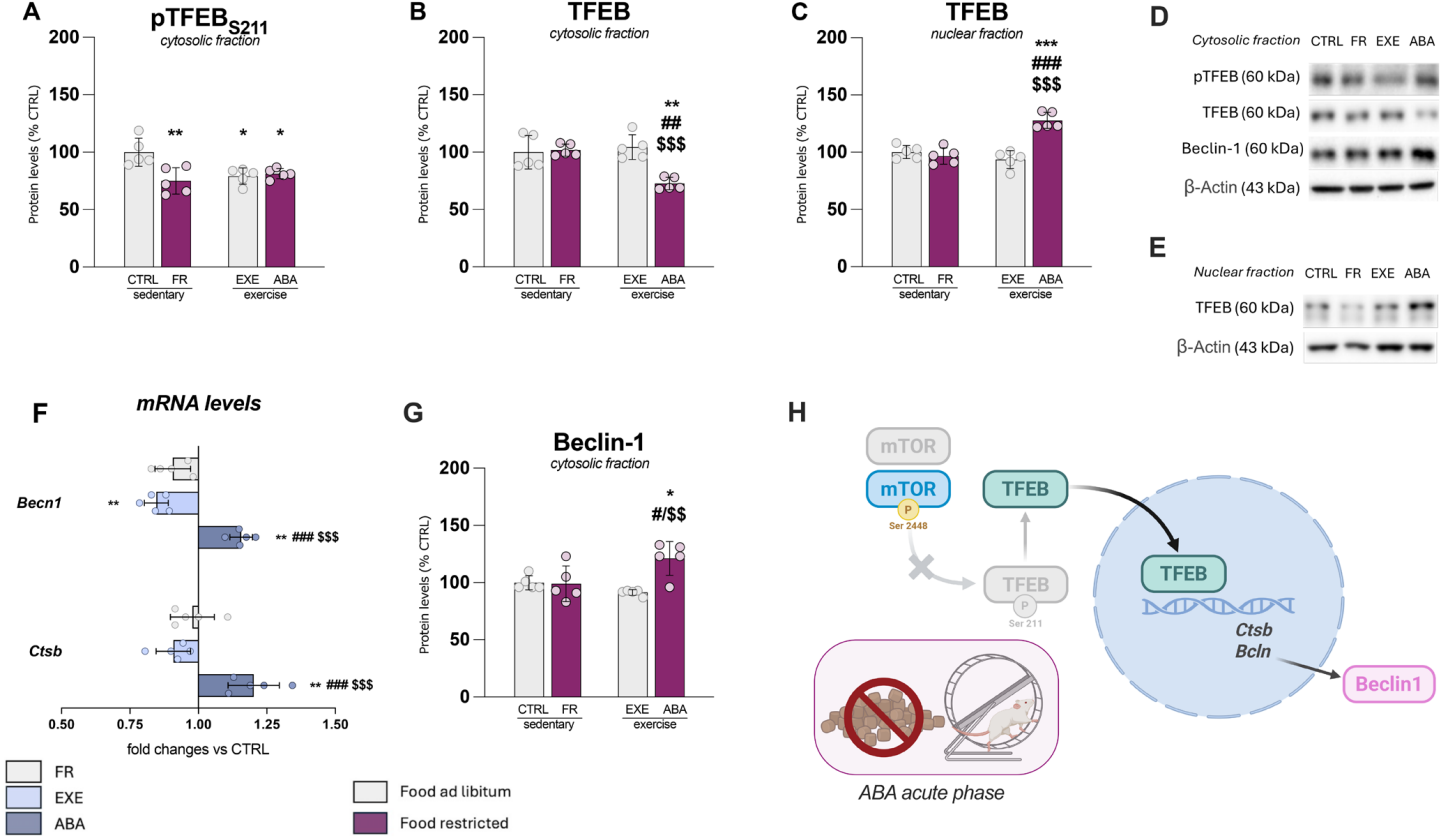
The induction of the ABA phenotype alters TFEB protein levels and TFEB‐dependent gene expression in the dorsal hippocampus at the acute phase. Panel (A) shows protein expression levels of pTFEB_Ser211_ in the cytosolic fraction of CTRL, FR, EXE, and ABA rats; whereas panel (B) and (C) display protein expression levels of TFEB in the cytosolic and nuclear fraction, respectively. *Cathepsin‐b* (*Ctsb*) and *Beclin‐1* (*Becn1*) mRNA levels are represented in panel (F) and expressed as fold changes. Protein levels of Beclin‐1 are presented in panel (G). Representative immunoblots for each protein are shown in panels (D) and (E). Panel (H) provides a graphical representation of the autophagy molecular cascade in the ABA group, highlighting the targets described in the adjacent panels: colored elements indicate pathway activation; gray elements represent inactive components. Phosphorylation of mTOR usually prevents TFEB translocation by phosphorylating TFEB at S211. In ABA rats, enhanced mTOR phosphorylation is uncoupled from TFEB phosphorylation, resulting in TFEB translocation and TFEB‐mediated gene transcription. Data are expressed as percentage (%) of CTRL and represent the mean ± SD of each group. Two‐way ANOVA followed by Tukey's multiple comparison test *p < 0.05, **p < 0.01, ***p < 0.001 versus CTRL; ^#^ *p* < 0.05, ^##^ *p* < 0.01, ^###^ *p* < 0.001 versus FR; ^$$^ *p* < 0.01, ^$$$^ *p* < 0.001 versus EXE. ABA, activity‐based anorexia (*n* = 5); CTRL, control (*n* = 5); EXE, exercise (*n* = 5); FR, food restricted (*n* = 5).

**FIGURE 3:**
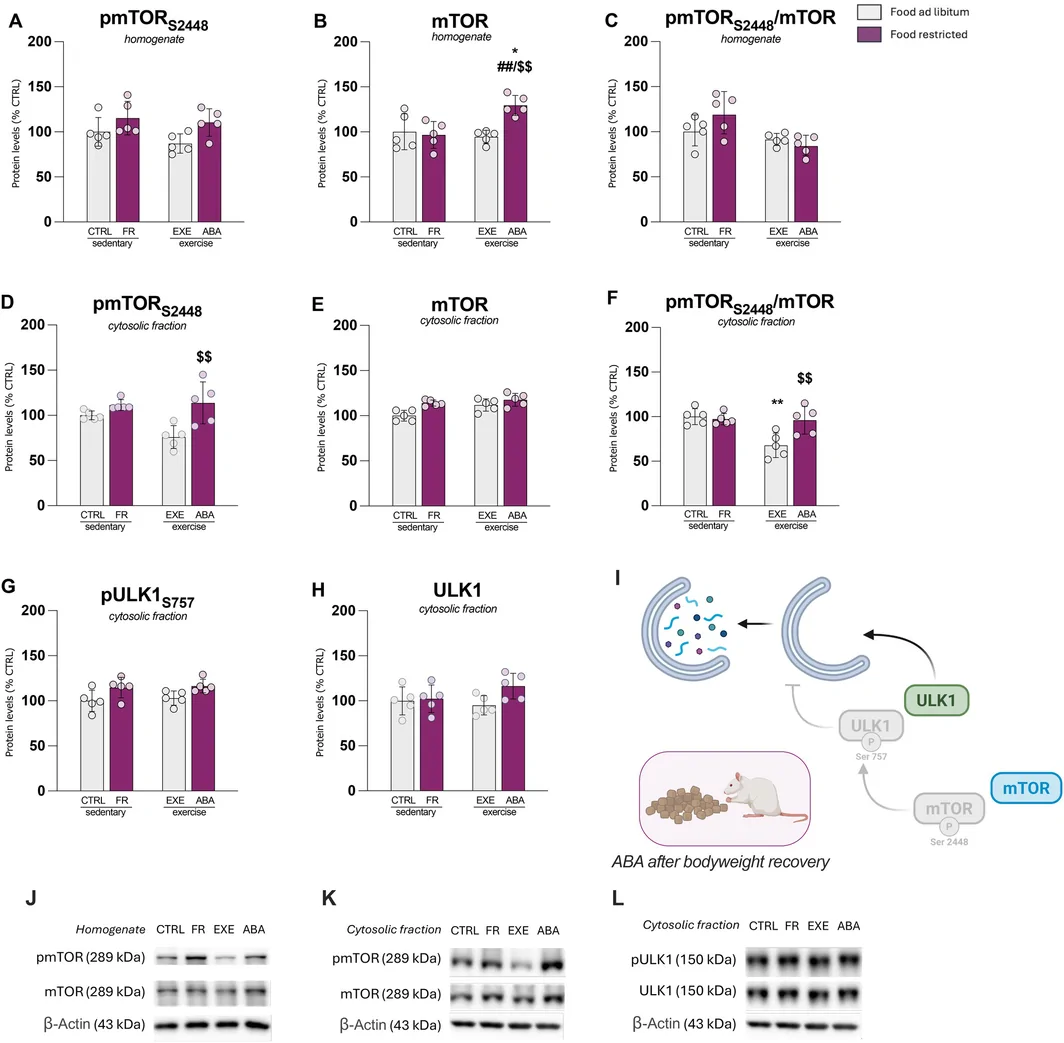
The induction of the ABA phenotype alters mTOR and ULK1 protein levels in the dorsal hippocampus after bodyweight recovery. Panels (A), (B), and (C) show pmTOR_Ser2448_, mTOR protein levels and the relative ratio pmTOR_Ser2448_/mTOR, respectively, in the whole homogenate of CTRL, FR, EXE and ABA rats, while panels (D), (E), and (F) display pmTOR_Ser2448,_ mTOR protein levels and the relative ratio pmTOR_Ser2448_/mTOR, respectively, in the cytosolic fraction. Panels (G) and (H) show protein pULK1_Ser757_ and ULK1 protein levels in the cytosolic fraction, respectively. Representative immunoblots for each protein are shown in panels (J–L). Panel (I) provides a graphical representation of the autophagy molecular cascade in the ABA group, highlighting the targets described in the adjacent panels: colored elements indicate pathway activation; gray elements represent inactive components. After bodyweight recovery, mTOR phosphorylation is comparable to CTRL levels, thereby preventing phosphorylation of ULK in S757 and allowing ULK‐mediated autophagy. Data are expressed as percentage (%) of CTRL and represent the mean ± SD (A, B, D–H) or the geometric mean ± geometric SD (C) of each group. Two‐way ANOVA followed by Tukey's multiple comparison test *p < 0.05, **p < 0.01 versus CTRL; ^##^ *p* < 0.01 versus FR; ^$$^ *p* < 0.01 versus EXE. ABA, activity‐based anorexia (*n* = 5); CTRL, control (*n* = 5); EXE, exercise (*n* = 5); FR, food restricted (*n* = 5).

**FIGURE 4:**
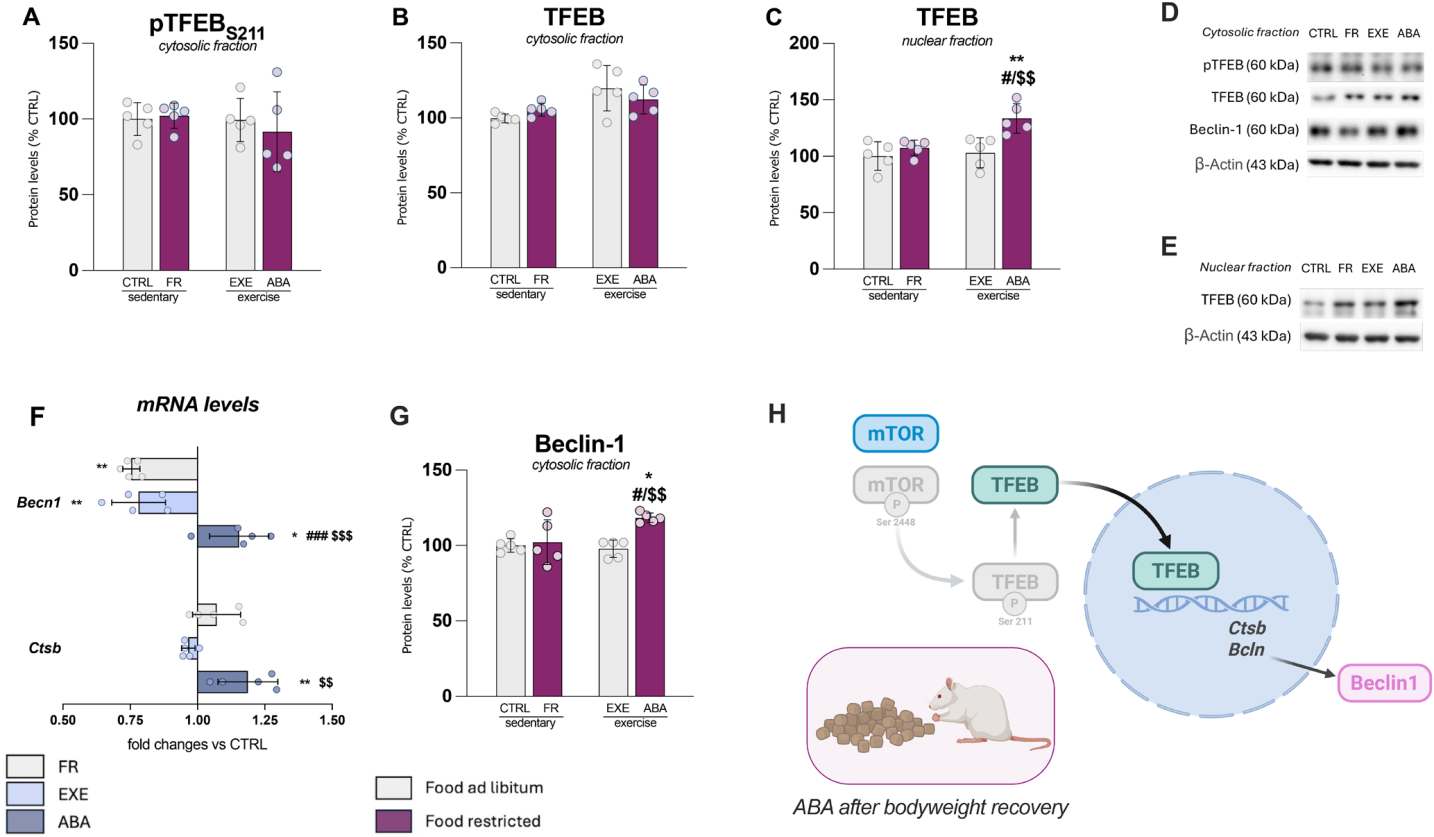
The induction of the ABA phenotype alters TFEB protein expression and TFEB‐dependent gene expression in the dorsal hippocampus after bodyweight recovery. Panel (A) shows protein expression levels of pTFEB_Ser211_ in the cytosolic fraction of CTRL, FR, EXE, and ABA rats; whereas panel (B) and panel (C) display protein expression levels of TFEB in the cytosolic and nuclear fraction, respectively. *Catepsin‐b* (*Ctsb*) and *Beclin‐1* (*Becn1*) mRNA levels are represented in panel (F) and expressed as fold changes. Protein levels of Beclin‐1 are presented in panel (G). Representative immunoblots for each protein are shown in panels (D) and (E). Panel (H) provides a graphical representation of the autophagy molecular cascade in the ABA group, highlighting the targets described in the adjacent panels: Colored elements indicate pathway activation; gray elements represent inactive components. After bodyweight recovery, mTOR and TFEB phosphorylation is comparable to CTRL levels, whereby allowing TFEB translocation and TFEB‐mediated gene transcription. Data are expressed as percentage (%) of CTRL and represent the mean ± SD of each group. Two‐way ANOVA followed by Tukey's multiple comparison test *p < 0.05, **p < 0.01 versus CTRL; ^#^ *p* < 0.05, ^###^ *p* < 0.001 versus FR; ^$$^ *p* < 0.01, ^$$$^ *p* < 0.001 versus EXE. ABA, activity‐based anorexia (*n* = 5); CTRL, control (*n* = 5); EXE, exercise (*n* = 5); FR, food restricted (*n* = 5).

**FIGURE 5:**
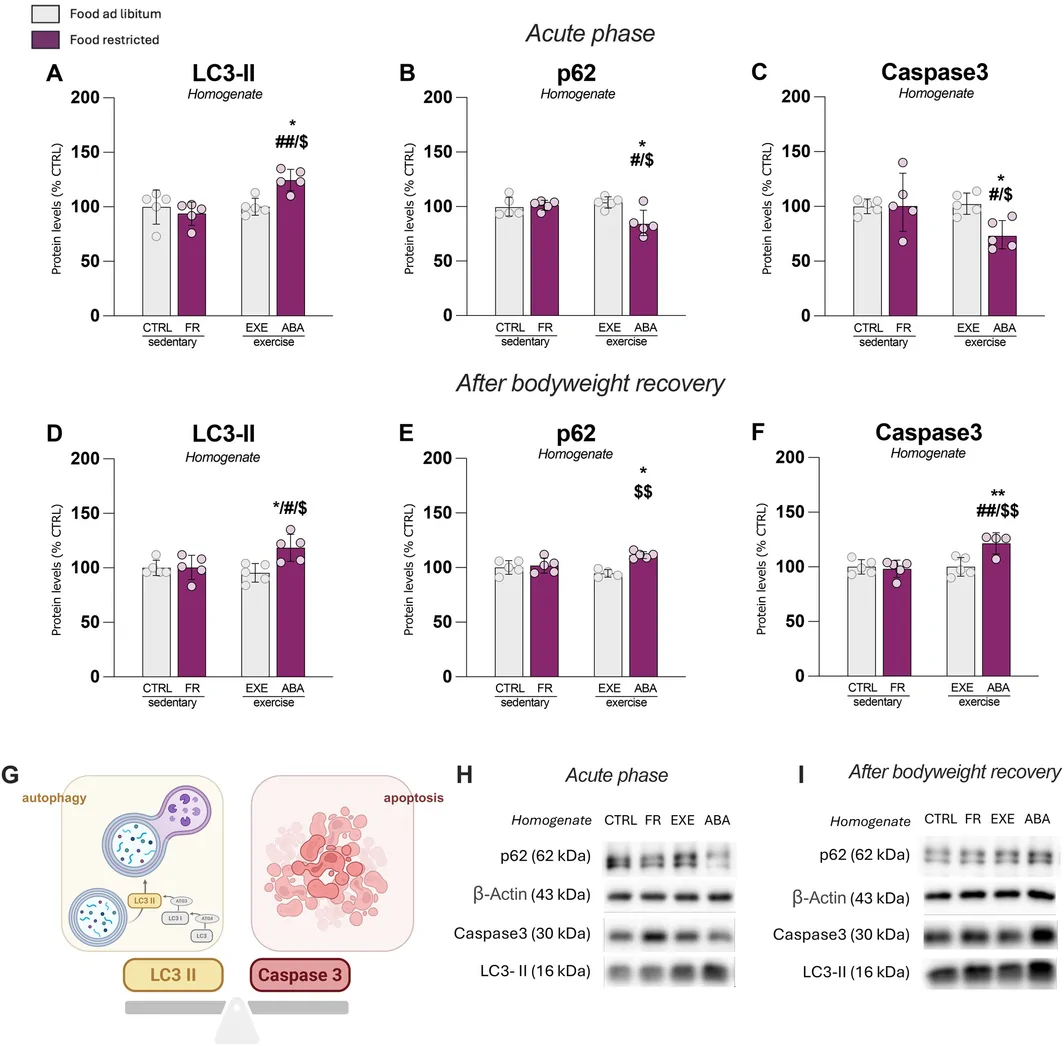
The induction of the ABA phenotype alters the protein expression of autophagic and apoptotic markers in the dorsal hippocampus at the acute phase and after bodyweight recovery. Panels (A) and (D) show protein expression levels of LC3‐II in the whole homogenate of CTRL, FR, EXE, and ABA rats at the acute phase and after bodyweight recovery, respectively. Panels (B) and (E) show protein expression levels of p62 in whole homogenate at the acute phase and after bodyweight recovery, respectively. Panels (C) and (F) show protein expression levels of Caspase3 in whole homogenate at the acute phase and after bodyweight recovery, respectively. Representative immunoblots for each protein are shown in panels (H) and (I), while panel (G) outlines the molecular involvement of LC3‐II and Caspase3 in cellular homeostasis and cell death mechanisms. Data are expressed as a percentage (%) of CTRL and represent the mean ± SD (A, B, D, E, F) or the geometric mean ± geometric SD (C) of each group. Two‐way ANOVA followed by Tukey's multiple comparison test *p < 0.05, **p < 0.01 versus CTRL; ^#^ *p* < 0.05, ^##^ *p* < 0.01 versus FR; ^$^ *p* < 0.05, ^$$^ *p* < 0.01 versus EXE. ABA, activity‐based anorexia (*n* = 5; panel F *n* = 4); CTRL, control (*n* = 5); EXE, exercise (*n* = 5; panel E *n* = 4); FR, food restricted (*n* = 5).

Detection of immunocomplexes was conducted using enhanced chemiluminescence (Cyanagen, Italia, Cat# XLS071P and XLS071L) and Chemidoc MP Imaging System (Bio‐Rad Laboratories, RRID: SCR_019037). Optical density (OD) of signals was analyzed with Image Lab software (Bio‐Rad Laboratories, RRID: SCR_014210). All image analyses were conducted in a blinded manner. The final results represent the average from at least two different gel runs, by means of a correction factor: correction factor gel B = average of (OD protein of interest/OD β‐actin for each CTRL sample loaded in gel A)/(OD protein of interest/OD β‐Actin for the same sample loaded in gel B) (Caffino et al. 2020). Full‐size original cropped immunoblots related to protein expression levels evaluated in the present study are enclosed in supplementary (Figures S2–S6) and representative immunoblots for each protein are shown in Figures 1J–L, 2D,E, 3J–L, 4D,E, 5H,I.

### 2.5 Statistical Analysis

Sample sizes (*n*) were determined based on prior studies using the same experimental model and effect size (effect size = 0.25, α error probability =0.05, and power = 0.8) calculated with the software G*power 3.1.9.2 (http://gpower.hhu.de/). Data were collected from individual animals (independent determinations). For each molecular target, normality of data distribution was assessed by D'Agostino‐Pearson omnibus normality test. When data were not normally distributed, they were log‐transformed (log_10_OD) and reassessed for normality. If the assumption of normality was still not met, outliers were detected with Grubbs test and removed from the specific target (final n per group is specified in each figure legend); normality was then assessed, as reported in Tables S1 and S6. Homogeneity of variance was assessed with the Brown‐Forsythe test (Tables S2 and S7). Given that the normality and the homogeneity of variance assumptions were met, molecular changes were analyzed using a two‐way ANOVA, with physical activity (sedentary vs. exercise) and food availability (ad libitum vs. restricted) as independent variables at the two time points, acute phase (PND42) and after bodyweight recovery (PND49). When significant interaction terms were observed, Tukey's multiple comparisons test was performed to identify differences between specific groups. Detailed statistical results, including F and *p* values for the main effects and interactions from the two‐way ANOVA, mean differences, 95% confidence intervals of differences and *p* values of post hoc comparisons are provided in Tables S3–S5, S8, and S9. Data are presented as mean % ± standard deviations (SD) versus CTRL group or as geometric mean % ± geometric SD versus CTRL group, if the dataset was analyzed using the logarithmic normalization. All statistical analyses were conducted using Prism 9.5.0 (GraphPad Software, San Diego, CA, USA, RRID:SCR_002798), with significance assumed at *p* < 0.05.

## 3 Results

We previously demonstrated that food restriction exacerbates physical activity on a wheel, leading to the development of the AN‐like phenotype in ABA rats. The peak of weight loss was established at PND42, representative of the acute phase of the pathology, with ABA animals showing lower bodyweight compared to all other groups and greater distance traveled (Mottarlini et al. 2022).

To evaluate whether ABA induction altered the autophagic processes in the dHip, we investigated the expression and cellular localization of key determinants of the autophagic machinery. We first analyzed mTOR phosphorylation and expression, a key actor of the autophagic machinery, in the dHip at the acute phase. In the homogenate, pmTOR_S2448_ levels were significantly increased only in the ABA group (Figure 1A), whereas total mTOR protein levels remained unchanged across groups (Figure 1B). When looking at the ratio of pmTOR_S2448_ to total mTOR, as an index of activation, no differences were observed across groups (Figure 1C). Similarly, in the cytosolic fraction, mTOR phosphorylation in S2448 was increased only in the ABA group (Figure 1D). Instead, the expression of mTOR in the cytosolic fraction was reduced only in the ABA rats (Figure 1E). These molecular changes resulted in an increase of pmTOR_S2448_/mTOR ratio only in the ABA group (Figure 1F). Besides mTOR phosphorylation, we also observed a concomitant increase of ULK phosphorylation at the inhibitory site S757 (Figure 1G) and of its total form only in the ABA group (Figure 1H), suggesting that the induction of the AN phenotype may inhibit the initiation of the autophagic machinery.

In parallel, we investigated the role of TFEB‐mediated transcriptional regulation of autophagy, fundamental for the biogenesis of the autophagosomes and lysosomal fusion, assessing the phosphorylation levels of TFEB in S211 and its total form in the cytosolic fraction. We found reduced TFEB phosphorylation in S211 in all the experimental groups (Figure 2A), while the total form was reduced only in ABA rats with respect to the other groups (Figure 2B). When activated (e.g., during starvation or cellular stress), TFEB translocates into the nucleus where it functions as a transcription factor (Napolitano and Ballabio 2016). Accordingly, TFEB expression in the nucleus was increased only in the ABA rats (Figure 2C), therefore supporting decreased cytosolic retention and increased nuclear translocation. As a readout of TFEB transcriptional activity, we evaluated the expression of downstream target genes involved in autophagy and lysosomal formation, as *Becn1*, which codes for Beclin‐1, a protein involved in the biogenesis of autophagosomes (Tran et al. 2021), and *Ctsb*, which codes for Cathepsin B, a cysteine protease primarily found in lysosomes (Xie et al. 2023). At the acute phase, mRNA levels of *Becn1* were downregulated in EXE rats, whilst upregulated only in ABA group (Figure 2F) This increase was accompanied by elevated *Ctsb* levels (Figure 2F) and increased cytosolic Beclin‐1 protein expression (Figure 2G), in line with the increased TFEB nuclear localization. These results suggest enhanced autophagy induction and point toward an activation of TFEB‐associated autophagy under the condition of food restriction and hyperactivity, which may be independent of mTOR phosphorylation (Palmieri et al. 2017; Settembre et al. 2011).

After bodyweight recovery, we observed a different picture: in the total homogenate no changes were found in pmTOR_S2448_ (Figure 3A), while its total form was increased only in the ABA group (Figure 3B). Overall, no changes in the activation of mTOR expressed as ratio pmTOR_S2448_/mTOR were observed (Figure 3C). Conversely, in the cytosolic fraction, mTOR phosphorylation levels were elevated only in the ABA group (Figure 3D) in respect to the EXE group, while no changes were observed in mTOR total protein expression (Figure 3E). In line, the pmTOR_S2448_/mTOR ratio was enhanced in ABA rats only compared to EXE animals, which showed a lower ratio compared to CTRL (Figure 3F). Of note, ULK phosphorylation in S757 and its total protein levels (Figure 3G,H) were unaltered in ABA rats, thus turning off the inhibition of the autophagic machinery triggered by ULK phosphorylation in S757 in the acute phase.

Notably, in recovered rats, pTFEB_S211_ and its total cytosolic form did not show any changes (Figure 4A,B), while the nuclear TFEB expression was persistently increased in the ABA group (Figure 4C). While *Becn1* mRNA levels were reduced in FR and EXE rats, the ABA group showed a sustained upregulation of *Becn1* and *Ctsb* gene expression (Figure 4F) and Beclin‐1 protein levels (Figure 4G), pointing to a prolonged activation of the autophagic machinery only in ABA rats, despite bodyweight recovery and nutrient availability.

Lastly, we examined microtubule‐associated protein 1A/1B‐light chain 3 (LC3‐II) protein expression, a well‐established autophagosome marker (Mizushima et al. 2010), and p62, a widely studied autophagy receptor and an autophagy substrate itself (Komatsu et al. 2007; W. J. Liu et al. 2016), as additional indexes of the autophagic machinery. At the acute phase, only the ABA animals showed an increase in LC3‐II levels (Figure 5A) coupled with a reduction in p62 protein levels (Figure 5B). Instead, after bodyweight recovery, the increase in LC3‐II levels in ABA rats (Figure 5D) was paralleled by an enhanced expression of p62 in respect to CTRL and EXE groups (Figure 5E).

To explore the balance between autophagy and apoptosis, as these processes can occur in a consequential order and mutually regulate each other determining cells' fate (Mariño et al. 2014), we measured total Caspase3 as a pro‐apoptotic marker. In the acute phase, Caspase3 protein levels were reduced exclusively in the ABA group (Figure 5C), supporting a prevalent activation of autophagic over apoptotic mechanisms at the peak of the AN‐like phenotype. On the contrary, after bodyweight recovery, Caspase3 levels were increased only in the ABA rats (Figure 5F), indicating a potential transition toward a pro‐apoptotic response in the recovery phase.

## 4 Discussion

Our data show that ABA induction in adolescent female rats disrupts mTOR‐mediated autophagy in the dHip, leading to prolonged and enhanced mTOR‐independent and TFEB‐related autophagic mechanisms that persist even after 7 days of bodyweight recovery. When animals develop the AN phenotype, the increased expression of autophagic markers might serve as a survival mechanism to provide energy in a nutrient‐deprived and high‐energy demanding condition; however, its persistence after bodyweight recovery, when the need to activate these processes has ceased, might become deleterious. This potentially contributes to hippocampal dysfunctions previously observed in ABA rats (Chowdhury, Barbarich‐Marsteller, et al. 2014; Mottarlini et al. 2024; Targa et al. 2024) and in subjects with AN (Collantoni et al. 2021; Kappou et al. 2021; Myrvang et al. 2018).

AN, described by a GWAS study as a metabo‐psychiatric disorder (Watson et al. 2019), is characterized by an extreme imbalance between energy supply and demand, due to low caloric intake combined with hyperactivity, a condition that might contribute to rewiring cognitive domains, thus sustaining aberrant behaviors. One of the molecular mechanisms that could help the system overcome the energetic disequilibrium during food restriction in the short‐term is the activation of autophagic mechanisms, as previously observed in the hypothalamus of ABA mice (Kaushik et al. 2011; Nobis et al. 2018) and in the dHip of ABA rats in the present study. At the molecular level, we observed impaired autophagic mechanisms specifically in the ABA group, whereas no changes were detected when the effects of food restriction and exercise were examined separately. In fact, in the dHip of FR rats, despite 5 days of food restriction, we did not observe any changes in mTOR‐mediated autophagy or in general markers of autophagy, such as LC3‐II and p62. Although the lack of detectable changes in autophagic machinery under fasting conditions may appear inconsistent with previous findings on malnutrition, this result may reflect the ability of peripheral organs, such as the liver, to undergo autophagic degradation and supply nutrients to the brain during short periods of food deprivation. This mechanism may help preserve brain homeostasis, making the brain one of the last organs to experience nutrient deficiency (Mizushima et al. 2004; Nikoletopoulou and Tavernarakis 2018). Alternatively, the absence of alterations in FR rats may be due to the lack of substantial energy expenditure, since these rats do not engage in physical activity (He 2022). Peripheral organs involved in metabolic regulation may also account for the lack of autophagy‐related mechanisms in the brain of animals that performed only exercise (He et al. 2012). Moreover, studies addressing this topic have employed different regimens of nutrient deprivation or exercise, and the precise contribution of their duration to the activation of maladaptive versus adaptive autophagy remains unclear (Shabkhizan et al. 2023).

At the acute phase, ABA animals showed increased pmTOR_S2448_ and pULK1_S757_ levels in the cytosol, effects that may suggest a reduction in autophagic activity. These findings appear to contrast with previous reports showing that under starvation, dephosphorylation of mTOR at S2448 reduces mTOR‐mediated phosphorylation of ULK1, thus promoting autophagy to preserve brain function (Alers et al. 2012; Kim et al. 2011; Settembre et al. 2012). However, in our experimental condition, despite the increase in mTOR phosphorylation should point to a negative regulation of autophagy, TFEB nuclear translocation was enhanced, as indicated by reduced cytosolic and increased nuclear TFEB levels, as well as by enhanced *Becn1* and *Ctsb* mRNA levels, autophagy‐related genes whose transcription can be mediated by TFEB, and Beclin‐1 protein levels, a key regulator of autophagosome formation (Kang et al. 2011). Together, these findings point to a robust activation of autophagy under the combined conditions of food restriction and hyperactivity potentially associated with mechanisms involving TFEB and independent from mTOR phosphorylation. These mechanisms may be activated by other upstream regulators, such as calcineurin, AMPK and reactive oxygen species (Palmieri et al. 2017; Tong and Song 2015; X. Zhang et al. 2016). This suggests that, while mTOR signaling attempts to suppress autophagy at the achievement of the anorexic phenotype via ULK1, a TFEB‐associated mechanism, potentially through a mTOR‐independent regulation, remains active, indicating a dysregulated or unbalanced control of autophagic regulation. Conversely, the increased phosphorylation of mTOR and ULK1 may represent a compensatory mechanism secondary to TFEB‐related mechanisms aimed at limiting excessive expression of autophagic determinants, which could be mediated by TFEB, and thus preserving cellular homeostasis in the dHip. Indeed, it cannot be excluded that at earlier time points mTOR and ULK1 phosphorylation might be reduced as part of the physiological response to starvation. In conditions of high energetic demand not adequately sustained by nutrient availability, as those induced by the ABA paradigm, autophagy is likely required to sustain cellular energy balance and survival, suggesting that mTOR‐mediated inhibition of the pathway might be maladaptive. Conversely, maintaining autophagic mechanisms under persistent energy deficit might further sustain the aberrant hyperactive phenotype and ultimately contribute to cellular stress or damage.

To understand whether the impairment of autophagy was a transient strategy to cope with the peak of energy expenditure corresponding to the acute phase of the AN phenotype, the pathway was also investigated after one week of bodyweight recovery with standard food availability and no possibility to engage in physical activity. As in the acute phase, only ABA rats exhibited increased pmTOR_S2448_, suggesting enhanced mTOR activation in animals with a history of AN, which may reflect an attempt to restore cellular homeostasis in response to metabolic stress induced by food restriction and hyperactivity. However, other markers of autophagy, such as nuclear TFEB, *Ctsb* and *Becn1*, Beclin‐1, LC3‐II and p62 levels remained elevated, a condition that could lead to significant aberrant implications, including disruption of neuronal function and plasticity. Indeed, chronic stimulation of autophagic mechanisms in the hippocampus might impair synaptic maintenance and neurogenesis (Birdsall and Waites 2019; K. Zhang et al. 2023), processes essential for cognitive functions and memory consolidation and found altered in ABA rats (Barbarich‐Marsteller et al. 2013; Cao et al. 2025; Mottarlini et al. 2024; Targa et al. 2024) and in individuals with AN (Foerde and Steinglass 2017; Guardia et al. 2013). Thus, the persistently increased expression of autophagy markers observed in the ABA model after a week of weight recovery, in parallel with the lasting reduction of the leptin rCeceptor signaling that we observed in the dHip of ABA rats (Targa et al. 2024), might have significant implications for hippocampal function. Autophagy, while initially neuroprotective, may contribute to neuronal damage and cognitive deficits when overactivated. The sustained autophagy even after weight recovery might participate to the impaired hippocampal‐dependent memory, which we have previously demonstrated in ABA rats both in the acute phase and after recovery by means of the Spatial Object Recognition test (Mottarlini et al. 2024). Despite no direct correlation can be drawn because of the different cohorts of animals involved and despite we formerly studied the whole hippocampus, without dissecting between its dorsal and ventral subregions, we can speculate that the enhanced autophagy within the dHip may also sustain, at least in part, the molecular rearrangement of the synapses observed in the above‐mentioned study. This hypothesis is also supported by the reduced expression of the Collapsin response mediator protein 2 (CRMP2) levels, a marker of cytoskeletal stability, in the dHip (Figure S1) and the dysregulation of hippocampal BDNF‐mediated neuroplastic processes (Mottarlini et al. 2025), as emerging evidence is unveiling a role for autophagy in degrading AMPA receptors, scaffolding proteins and cytoskeletal components and in modulating synaptic pruning (Glatigny et al. 2019; Shehata et al. 2012; Tang et al. 2014).

Uncontrolled autophagy is known to result in excessive degradation of essential cellular components, initiating a cascade of events that can ultimately lead to apoptosis (Maiuri et al. 2007). At the acute phase of the pathology, we observed an increase in autophagy, as indicated by enhanced LC3‐II levels and reduced p62 levels (W. J. Liu et al. 2016), whereas pro‐apoptotic signaling, measured as Caspase3 levels, was reduced. This suggests that the herein‐studied mechanism has not resulted in the degradation of vital cellular components. However, prolonged and uncontrolled autophagy may progressively shift toward a pro‐apoptotic response after recovery. Of note, the parallel increase in LC3‐II and p62 after weight recovery may result from improper degradation, indicating impaired or dysfunctional autophagic mechanisms (Mathew et al. 2009; Runwal et al. 2019). Moreover, p62 accumulation has been suggested to promote the transition toward apoptosis by serving as a signaling factor for caspase activation (Moscat and Diaz‐Meco 2009), a mechanism that may be consistent with the enhanced Caspase3 levels observed in the ABA animals following the 7‐day period of recovery. This dynamic interplay between autophagy and apoptosis highlights the potentially deleterious consequences of a chronic enhanced autophagic activity, which shifts from an adaptive coping strategy to overcome nutrient deficiency at the acute phase of AN toward a driver of potential cellular death during the recovery phase, an aspect that warrants further investigation.

The findings of this study have to be seen in light of some limitations. Despite a higher rate of incidence in the female population, cases among males are increasing (Gorrell and Murray 2019; Mitchison and Mond 2015); here we used only female rats limiting the generalizability of our findings to a broader population. Second, we investigated this mechanism only in the dHip; accordingly, future studies should investigate whether these mechanisms are conserved in other brain regions or whether the observed changes may be subregion specific. Third, without a pharmacologic treatment it is difficult to establish a causal link between autophagic mechanisms, ABA phenotype and hippocampal‐related behavioral deficits; however, our results are encouraging and may pave the way for more targeted therapeutic strategies. Finally, the 7‐day recovery phase examined in this study models the acute weight restoration, which represents the primary initial goal in clinical practice. Although our findings indicate that weight recovery does not necessarily imply biological recovery and, therefore, full remission, whether these alterations persist over longer periods or eventually resolve remains an open question that warrants further investigation.

## 5 Conclusions

Our findings provide valuable insights into the potential mechanisms underlying cognitive deficits and maladaptive behaviors in AN. Persistent enhanced autophagic molecular determinants in the dHip may serve as a dysfunctional response, initially aimed at adaptation to starvation but ultimately leading to apoptosis, potentially contributing to hippocampal damage and cognitive impairments. This maladaptive autophagic response may also increase cellular senescence (Singh et al. 2026) and vulnerability to psychiatric comorbidities, such as anxiety and depression (Brivio et al. 2025), commonly observed in patients with AN.

Future studies should aim at unveiling the intricate balance between autophagy and apoptosis in the hippocampus and at investigating the causal relationships between autophagy dysregulation, cognitive deficits, and behavioral abnormalities.

## Author Contributions

**Beatrice Rizzi:** conceptualization, data curation, formal analysis, writing – review and editing, writing – original draft, methodology, validation. **Giorgia Targa:** conceptualization, writing – original draft, writing – review and editing, formal analysis, data curation, methodology, validation. **Sofia Taddini:** writing – review and editing, visualization, data curation, investigation. **Susanna Parolaro:** writing – review and editing, data curation, investigation. **Letizia Rapini:** writing – review and editing, investigation. **Fabio Fumagalli:** writing – review and editing, supervision. **Lucia Caffino:** project administration, supervision, data curation, writing – review and editing, writing – original draft, funding acquisition, conceptualization, validation, visualization. **Francesca Mottarlini:** conceptualization, funding acquisition, writing – review and editing, data curation, supervision, validation, visualization.

## Funding

This work was supported by Ministero dell'Istruzione, dell'Università e della Ricerca, P2022E4MLS, Progetto Eccellenza 2023–2027; Fondazione Cariplo, 2023–1003; Università degli Studi di Milano, Piano di Sostegno alla Ricerca.

## Conflicts of Interest

The authors declare no conflicts of interest.

## Supporting information


**Table S1:** Detailed statistics of D'Agostino‐Pearson omnibus normality test related to protein levels measured in the dorsal hippocampus of CTRL, FR, EXE, and ABA rats at the establishment of the acute phase and after a seven‐day recovery period and presented in Figures 1, 2, 3, 4, 5 and in Figure S1. Statistical significance was considered when *p* < 0.05, for α = 0.05.
**Table S2:** Detailed statistics of Brown‐Forsythe test to assess homogeneity of variances related to protein levels measured in the dorsal hippocampus of CTRL, FR, EXE and ABA rats at the establishment of the acute phase and after a seven‐day recovery period and presented in Figures 1, 2, 3, 4, 5 and in Figure S1. Statistical significance was considered when *p* < 0.05, for α = 0.05.
**Table S3:** Detailed F values, degrees of freedom and *p* values relative to two‐way ANOVA analyses of protein levels measured in the dorsal hippocampus of CTRL, FR, EXE and ABA rats at the establishment of the acute phase and after a seven‐day recovery period and presented in Figures 1, 2, 3, 4, 5 and in Figure S1. Statistical significance was considered when *p* < 0.05, for α = 0.05.
**Table S4:** Detailed mean differences (diff.), 95% confidence interval (CI) of difference and *p* values relative to Tukey's multiple comparisons test following significant interaction of two‐way ANOVA analyses of protein levels measured in the dorsal hippocampus of CTRL, FR, EXE, and ABA rats at the establishment of the acute phase and presented in Figures 1, 2, 3, 4, 5 and in Figure S1. Statistical significance was considered when *p* < 0.05, for α = 0.05.
**Table S5:** Detailed mean differences (diff.), 95% Confidence Interval (CI) of difference and *p* values relative to Tukey's multiple comparisons test following significant interaction of two‐way ANOVA analyses of protein levels measured in the dorsal hippocampus of CTRL, FR, EXE, and ABA rats after a seven‐day recovery period and presented in Figures 1, 2, 3, 4, 5. Statistical significance was considered when *p* < 0.05, for α = 0.05.
**Table S6:** Detailed statistics of D'Agostino‐Pearson omnibus normality test related to mRNA levels evaluated in the dorsal hippocampus of CTRL, FR, EXE, and ABA rats at the establishment of the acute phase and after a seven‐day recovery period and presented in Figures 2 and 4. Statistical significance was considered when *p* < 0.05, for α = 0.05.
**Table S7:** Detailed statistics of Brown‐Forsythe test to assess homogeneity of variances related to mRNA levels evaluated in the dorsal hippocampus of CTRL, FR, EXE, and ABA rats at the establishment of the acute phase and after a seven‐day recovery period and presented in Figures 2 and 4. Statistical significance was considered when *p* < 0.05, for α = 0.05.
**Table S8:** Detailed F values, degrees of freedom and *p* values relative to two‐way ANOVA analyses of mRNA levels evaluated in the dorsal hippocampus of CTRL, FR, EXE, and ABA rats at the establishment of the acute phase and after a seven‐day recovery period and presented in Figures 2 and 4. Statistical significance was considered when *p* < 0.05, for α = 0.05.
**Table S9:** Detailed mean differences (diff.), 95% Confidence Interval (CI) of difference and *p* values relative to Tukey's multiple comparisons test following significant interaction of two‐way ANOVA analyses of mRNA levels evaluated in the dorsal hippocampus of CTRL, FR, EXE, and ABA rats at the establishment of the acute phase and after a seven‐day recovery period and presented in Figures 2 and 4. Statistical significance was considered when *p* < 0.05, for α = 0.05.
**Figure S1:** The induction of the ABA phenotype reduces CRMP2 protein levels in the dorsal hippocampus at the acute phase. Panels (A) and (B) show CRMP2 protein levels in the whole homogenate of CTRL, FR, EXE, and ABA rats, at the acute phase and after seven days of bodyweight recovery, respectively. Representative immunoblots for each protein are shown in panel (C). Data are expressed as percentage (%) of CTRL and represent the mean ± SD. Two‐way ANOVA followed by Tukey's multiple comparison test *p < 0.05 versus CTRL; #*p* < 0.05 versus FR; $ *p* < 0.05 versus EXE. CTRL: control (*n* = 5); FR: food restricted (*n* = 5); EXE: exercise (*n* = 5); ABA: activity‐based anorexia (*n* = 5).
**Figure S2:** Example of full‐size cropped immunoblots related to the protein expression levels of (A) pmTOR_S2448_ (289 kDa), mTOR (289 kDa), β‐actin (43 kDa), Caspase3 (30 kDa), LC3‐II (16 kDa) measured in the whole homogenate of the dorsal hippocampus; (B) pmTOR_S2448_ (289 kDa), mTOR (289 kDa), pULK1_S757_ (150 kDa), ULK1 (150 kDa), pTFEB_S211_ (60 kDa), TFEB (60 kDa), Beclin‐1 (60 kDa), β‐actin (43 kDa) in the cytosolic fraction of the dorsal hippocampus; (C) TFEB (60 kDa) and β‐actin (43 kDa) in the nuclear fraction of female rats at the acute phase of the anorexic phenotype (PND42), and presented in Figures 1, 2 and 5. Underlined lanes indicate the representative blots shown in the related figures. 1: CTRL: controls, 2: FR: food restricted, 3: EXE: exercise, 4: ABA: Activity‐based anorexia.
**Figure S3:** Example of full‐size cropped immunoblots related to the protein expression levels of (A) pmTOR_S2448_ (289 kDa), mTOR (289 kDa), β‐actin (43 kDa), Caspase3 (30 kDa), LC3‐II (16 kDa) measured in the whole homogenate of the dorsal hippocampus; (B) pmTOR_S2448_ (289 kDa), mTOR (289 kDa), pULK1_S757_ (150 kDa), ULK1 (150 kDa), pTFEB_S211_ (60 kDa), TFEB (60 kDa), Beclin‐1 (60 kDa), β‐actin (43 kDa) in the cytosolic fraction of the dorsal hippocampus; (C) TFEB (60 kDa) and β‐actin (43 kDa) in the nuclear fraction of female rats after seven days of bodyweight recovery (PND49), and presented in Figures 3, 4, 5. Underlined lanes indicate the representative blots shown in the related figures. 1: CTRL: controls, 2: FR: food restricted, 3: EXE: exercise, 4: ABA: Activity‐based anorexia.
**Figure S4:** Example of merged images of the membranes showing the labeled protein ladder and the cropped immunoblot related to the protein expression levels of (A) pmTOR_S2448_ (289 kDa), mTOR (289 kDa), β‐actin (43 kDa), Caspase3 (30 kDa), LC3‐II (16 kDa) measured in the whole homogenate of the dorsal hippocampus; (B) pmTOR_S2448_ (289 kDa), mTOR (289 kDa), pULK1_S757_ (150 kDa), ULK1 (150 kDa), pTFEB_S211_ (60 kDa), TFEB (60 kDa), Beclin‐1 (60 kDa), β‐actin (43 kDa) in the cytosolic fraction of the dorsal hippocampus; (C) TFEB (60 kDa) and β‐actin (43 kDa) in the nuclear fraction of female rats at the acute phase of the anorexic phenotype (PND42), and presented in Figures 1, 2 and 5. 1: CTRL: controls, 2: FR: food restricted, 3: EXE: exercise, 4: ABA: Activity‐based anorexia.
**Figure S5:** Example of merged images of the membranes showing the labeled protein ladder and the cropped immunoblot related to the protein expression levels of (A) pmTOR_S2448_ (289 kDa), mTOR (289 kDa), β‐actin (43 kDa), Caspase3 (30 kDa), LC3‐II (16 kDa) measured in the whole homogenate of the dorsal hippocampus; (B) pmTOR_S2448_ (289 kDa), mTOR (289 kDa), pULK1_S757_ (150 kDa), ULK1 (150 kDa), pTFEB_S211_ (60 kDa), TFEB (60 kDa), Beclin‐1 (60 kDa), β‐actin (43 kDa) in the cytosolic fraction of the dorsal hippocampus; (C) TFEB (60 kDa) and β‐actin (43 kDa) in the nuclear fraction of female rats after seven days of bodyweight recovery (PND49), and presented in Figures 3, 4, 5. 1: CTRL: controls, 2: FR: food restricted, 3: EXE: exercise, 4: ABA: Activity‐based anorexia.
**Figure S6:** Example of full‐size cropped immunoblots and merged images of membranes showing the labeled protein ladder and the cropped immunoblot related to the protein expression levels of (A), (B) CRMP2 (70 kDa), p62 (62 kDa) and β‐actin (43 kDa) measured in the whole homogenate of the dorsal hippocampus at the acute phase; (C), (D) CRMP2 (70 kDa), p62 (62 kDa), and β‐actin (43 kDa) in the whole homogenate of the dorsal hippocampus after seven days of bodyweight recovery and presented in Figure 5 and in Figure S1. Underlined lanes indicate the representative blots shown in the related figures. 1: CTRL: controls, 2: FR: food restricted, 3: EXE: exercise, 4: ABA: Activity‐based anorexia.
**Figure S7:** Merged images of the membrane showing labeled protein ladder and the cropped immunoblots related to the protein expression of β‐actin (43 kDa), GAPDH (36 kDa) and H3 (16 kDa) measured in the whole homogenate, cytosolic fraction and nuclear fraction of the dorsal hippocampus of CTRL animals, to assess successful subcellular fractionation. CTRL = controls.

## Data Availability

The data that support the findings of this study are available from the corresponding author upon reasonable request.
