# Supplementary material for: Dysregulation of Hippocampal Autophagic Machinery in the Activity‐Based Anorexia Rat Model
Source: J Neurochem. 2026 Sep 10;170(9):e70549. doi: 10.1111/jnc.70549 (PMC13559218; doi:10.1111/jnc.70549)
Supplement: Supplementary file 1 [file JNC-170-e70549-s001.pdf]

Dysregulation of hippocampal autophagic machinery in the activity-based anorexia rat model

Beatrice Rizzi[#1], Giorgia Targa[#1], Sofia Taddini[1], Susanna Parolaro[1], Letizia Rapini[1], Fabio Fumagalli[1], Lucia Caffino[1*] and Francesca Mottarlini[1]

[1] Department of Pharmacological and Biomolecular Sciences 'Rodolfo Paoletti', Università degli Studi di Milano, Via Balzaretti 9, 20133 Milano, Italy

[#] these authors equally contributed

*Corresponding author: Lucia Caffino, Department of Pharmacological and Biomolecular Sciences 'Rodolfo Paoletti', Università degli Studi di Milano, Via Balzaretti 9, 20133 Milano, Italy Phone +39-02-50318245; E-mail: lucia.caffino@unimi.it
ORCID: https://orcid.org/0000-0001-8045-3146

**Homogenate**	Acute phase		After recovery	
Normality test : D'Agostino-Pearson omnibus	**Statistics**	**P value**	**Statistics**	**P value**
$pmTOR_{S2448}$	4,614	0,0995	1,113	0,5731
mTOR	1,189	0,5517	0,7496	0,6874
$pmTOR_{S2448}$/mTOR	0,4166	0,8120	3,550	0,1695
LC3-II	5,601	0,0608	0,3340	0,8462
Caspase3	2,562	0,2778	1,831	0,4003
p62	5,916	0,0519	0,006330	0,9968
CRMP2	0,08410	0,9588	4,637	0,0984

**Cytosolic fraction**	Acute phase		After recovery	
Normality test : D'Agostino-Pearson omnibus	**Statistics**	**P value**	**Statistics**	**P value**
$pmTOR_{S2448}$	0,2108	0,9000	2,483	0,2889
mTOR	0,09635	0,9530	1,984	0,3708
$pmTOR_{S2448}$/mTOR	0,7214	0,6972	0,9400	0,6250
$pULK1_{S757}$	3,079	0,2145	0,4588	0,7950
ULK1	0,8218	0,6630	0,7933	0,6726
$pTFEB_{S211}$	0,9097	0,6345	3,097	0,2126
TFEB	1,997	0,3684	3,809	0,1489
Beclin-1	2,703	0,2589	3,263	0,1957

**Nuclear Fraction**	Acute phase		After recovery	
Normality test : D'Agostino-Pearson omnibus	**Statistics**	**P value**	**Statistics**	**P value**
TFEB	1,151	0,5624	1,264	0,5314

Table 1. Detailed statistics of D'Agostino-Pearson omnibus normality test related to protein levels measured in the dorsal hippocampus of CTRL, FR, EXE and ABA rats at the establishment of the acute phase and after a seven-day recovery period and presented in figures 1-5 and in supplementary figure 1. Statistical significance was considered when $p < 0.05$, for $\alpha = 0.05$

**Homogenate**	Acute phase		After recovery	
Brown-Forsythe test	**F (DFn, DFd)**	**P value**	**F (DFn, DFd)**	**P value**
pmTOR$_{S2448}$	0,3897 (3, 16)	0,7620	0,1845 (3, 16)	0,9054
mTOR	0,6476 (3, 16)	0,5958	0,9324 (3, 16)	0,4479
pmTOR$_{S2448}$/mTOR	0,4080 (3, 16)	0,7494	0,4996 (3, 16)	0,6878
LC3-II	0,2060 (3, 16)	0,8907	0,4550 (3, 16)	0,7174
Caspase3	1,324 (3, 16)	0,3012	0,09082 (3, 15)	0,9640
p62	0,5360 (3, 16)	0,6643	0,9703 (3, 15)	0,4326
CRMP2	0,6889 (3, 16)	0,5719	0,2022 (3, 16)	0,8933

**Cytosolic fraction**	Acute phase		After recovery	
Brown-Forsythe test	**F (DFn, DFd)**	**P value**	**F (DFn, DFd)**	**P value**
pmTOR$_{S2448}$	1,314 (3, 16)	0,3045	3,137 (3, 16)	0,0546
mTOR	0,9776 (3, 16)	0,4278	0,7509 (3, 16)	0,5377
pmTOR$_{S2448}$/mTOR	1,556 (3, 16)	0,2388	1,080 (3, 16)	0,3856
pULK1$_{S757}$	1,508 (3, 16)	0,2506	0,1634 (3, 16)	0,9194
ULK1	0,9380 (3, 16)	0,4453	0,1401 (3, 16)	0,9345
pTFEB$_{S211}$	0,7873 (3, 16)	0,5184	0,9002 (3, 16)	0,4627
TFEB	0,8507 (3, 16)	0,4864	2,316 (3, 16)	0,1145
Beclin-1	1,270 (3, 16)	0,3183	2,297 (3, 16)	0,1166

**Nuclear Fraction**	Acute phase		After recovery	
Brown-Forsythe test	**F (DFn, DFd)**	**P value**	**F (DFn, DFd)**	**P value**
TFEB	0,02580 (3, 16)	0,9942	0,5298 (3, 16)	0,6682

Table 2. Detailed statistics of Brown-Forsythe test to assess homogeneity of variances related to protein levels measured in the dorsal hippocampus of CTRL, FR, EXE and ABA rats at the establishment of the acute phase and after a seven-day recovery period and presented in figures 1-5 and in supplementary figure 1. Statistical significance was considered when $p < 0.05$, for $\alpha = 0.05$

**Homogenate**		Acute phase		After recovery	
Two-way ANOVA		**F value**	**P value**	**F value**	**P value**
pmTOR$_{S2448}$	Interaction	F (1, 16) = 4,607	P=0,0475	F (1, 16) = 0,3515	P=0,5615
	Exercise	F (1, 16) = 4,355	P=0,0533	F (1, 16) = 1,716	P=0,2087
	Food restriction	F (1, 16) = 6,654	P=0,0202	F (1, 16) = 7,975	P=0,0122
mTOR	Interaction	F (1, 16) = 0,4243	P=0,5240	F (1, 16) = 9,270	P=0,0077
	Exercise	F (1, 16) = 1,033	P=0,3245	F (1, 16) = 4,762	P=0,0443
	Food restriction	F (1, 16) = 2,238	P=0,1541	F (1, 16) = 6,189	P=0,0243
pmTOR$_{S2448}$/mTOR	Interaction	F (1, 16) = 5,402	P=0,0336	F (1, 16) = 3,447	P=0,0819
	Exercise	F (1, 16) = 1,582	P=0,2265	F (1, 16) = 10,44	P=0,0052
	Food restriction	F (1, 16) = 1,764	P=0,2027	F (1, 16) = 0,4862	P=0,4956
LC3-II	Interaction	F (1, 16) = 9,009	P=0,0085	F (1, 16) = 6,398	P=0,0223
	Exercise	F (1, 16) = 9,269	P=0,0077	F (1, 16) = 2,327	P=0,1466
	Food restriction	F (1, 16) = 3,303	P=0,0879	F (1, 16) = 6,718	P=0,0197
Caspase3	Interaction	F (1, 16) = 4,842	P=0,0428	F (1, 15) = 9,780	P=0,0069
	Exercise	F (1, 16) = 3,911	P=0,0654	F (1, 15) = 9,557	P=0,0074
	Food restriction	F (1, 16) = 4,750	P=0,0446	F (1, 15) = 7,019	P=0,0182
p62	Interaction	F (1, 16) = 7,395	P=0,0152	F (1, 15) = 9,094	P=0,0087
	Exercise	F (1, 16) = 2,922	P=0,1067	F (1, 15) = 0,7048	P=0,4144
	Food restriction	F (1, 16) = 5,521	P=0,0320	F (1, 15) = 14,09	P=0,0019
CRMP2	Interaction	F (1, 16) = 7,497	P=0,0146	F (1, 16) = 1,126	P=0,3045
	Exercise	F (1, 16) = 4,212	P=0,0569	F (1, 16) = 0,2498	P=0,6241
	Food restriction	F (1, 16) = 4,291	P=0,0549	F (1, 16) = 1,058	P=0,3190

**Cytosolic fraction**		Acute phase		After recovery	
Two-way ANOVA		**F value**	**P value**	**F value**	**P value**
pmTOR$_{S2448}$	Interaction	F (1, 16) = 11,74	P=0,0035	F (1, 16) = 4,709	P=0,0454
	Exercise	F (1, 16) = 12,31	P=0,0029	F (1, 16) = 3,122	P=0,0963
	Food restriction	F (1, 16) = 11,21	P=0,0041	F (1, 16) = 16,29	P=0,0010
mTOR	Interaction	F (1, 16) = 10,50	P=0,0051	F (1, 16) = 2,331	P=0,1463
	Exercise	F (1, 16) = 32,48	P<0,0001	F (1, 16) = 7,963	P=0,0123
	Food restriction	F (1, 16) = 34,42	P<0,0001	F (1, 16) = 13,44	P=0,0021
pmTOR$_{S2448}$/mTOR	Interaction	F (1, 16) = 18,66	P=0,0005	F (1, 16) = 8,602	P=0,0098
	Exercise	F (1, 16) = 27,04	P<0,0001	F (1, 16) = 10,21	P=0,0056
	Food restriction	F (1, 16) = 24,66	P=0,0001	F (1, 16) = 5,933	P=0,0269
pULK1$_{S757}$	Interaction	F (1, 16) = 17,96	P=0,0006	F (1, 16) = 0,01221	P=0,9134
	Exercise	F (1, 16) = 0,1468	P=0,7066	F (1, 16) = 0,2833	P=0,6018
	Food restriction	F (1, 16) = 33,63	P<0,0001	F (1, 16) = 10,06	P=0,0059
ULK1	Interaction	F (1, 16) = 11,83	P=0,0034	F (1, 16) = 2,351	P=0,1447
	Exercise	F (1, 16) = 0,4822	P=0,4974	F (1, 16) = 0,5702	P=0,4611
	Food restriction	F (1, 16) = 19,76	P=0,0004	F (1, 16) = 3,506	P=0,0795
pTFEB$_{S211}$	Interaction	F (1, 16) = 10,18	P=0,0057	F (1, 16) = 0,4687	P=0,5034
	Exercise	F (1, 16) = 2,879	P=0,1091	F (1, 16) = 0,5620	P=0,4643
	Food restriction	F (1, 16) = 7,439	P=0,0149	F (1, 16) = 0,1532	P=0,7007
TFEB	Interaction	F (1, 16) = 14,63	P=0,0015	F (1, 16) = 2,419	P=0,1394
	Exercise	F (1, 16) = 7,617	P=0,0139	F (1, 16) = 10,10	P=0,0058
	Food restriction	F (1, 16) = 11,66	P=0,0035	F (1, 16) = 0,05801	P=0,8127
Beclin-1	Interaction	F (1, 16) = 9,435	P=0,0073	F (1, 16) = 6,136	P=0,0248
	Exercise	F (1, 16) = 1,782	P=0,2006	F (1, 16) = 3,525	P=0,0788
	Food restriction	F (1, 16) = 8,554	P=0,0099	F (1, 16) = 9,403	P=0,0074

**Nuclear Fraction**		Acute phase		After recovery	
Two-way ANOVA		**F value**	**P value**	**F value**	**P value**
TFEB	Interaction	F (1, 16) = 35,53	P<0,0001	F (1, 16) = 4,904	P=0,0417
	Exercise	F (1, 16) = 15,44	P=0,0012	F (1, 16) = 7,780	P=0,0131
	Food restriction	F (1, 16) = 23,26	P=0,0002	F (1, 16) = 13,08	P=0,0023

Table 3. Detailed F values, degrees of freedom and p values relative to two-way ANOVA analyses of protein levels measured in the dorsal hippocampus of CTRL, FR, EXE and ABA rats at the establishment of the acute phase and after a seven-day recovery period and presented in figures 1-5 and in supplementary figure 1. Statistical significance was considered when $p < 0.05$, for $\alpha = 0.05$.

Homogenate		Acute phase		
Tukey's multiple comparisons test		**Mean diff.**	**95% CI of diff.**	**P value**
$pmTOR_{S2448}$	CTRL vs ABA	-0,1616	-0,3018 to -0,02149	0,0211
	FR vs. ABA	-0,1466	-0,2868 to -0,006482	0,0387
	EXE vs. ABA	-0,1637	-0,3038 to -0,02355	0,0194
LC3-II	CTRL vs. ABA	-0,2271	-0,4161 to -0,03810	0,0160
	FR vs. ABA	-0,2824	-0,4714 to -0,09342	0,0029
	EXE vs. ABA	-0,2251	-0,4141 to -0,03610	0,0170
Caspase3	CTRL vs. ABA	0,1364	0,003646 to 0,2691	0,0430
	FR vs. ABA	0,1370	0,004335 to 0,2698	0,0418
	EXE vs. ABA	0,1437	0,01095 to 0,2764	0,0316
p62	CTRL vs. ABA	0,1563	0,0004996 to 0,3121	0,0491
	FR vs. ABA	0,1705	0,01474 to 0,3264	0,0295
	EXE vs. ABA	0,1952	0,03940 to 0,3510	0,0119
CRMP2	CTRL vs. ABA	0,1170	0,002201 to 0,2317	0,0450
	FR vs. ABA	0,1359	0,02111 to 0,2506	0,0177
	EXE vs. ABA	0,1364	0,02165 to 0,2512	0,0173

Cytosolic fraction		Acute phase		
Tukey's multiple comparisons test		**Mean diff.**	**95% CI of diff.**	**P value**
$pmTOR_{S2448}$	CTRL vs. ABA	-0,1344	-0,2136 to -0,05509	0,0009
	FR vs. ABA	-0,1359	-0,2152 to -0,05661	0,0008
	EXE vs. ABA	-0,1327	-0,2120 to -0,05347	0,0010
mTOR	CTRL vs. ABA	0,07469	0,04856 to 0,1008	<0,0001
	FR vs. ABA	0,05773	0,03160 to 0,08386	<0,0001
	EXE vs. ABA	0,05882	0,03269 to 0,08495	<0,0001
$pmTOR_{S2448}$/mTOR	CTRL vs. ABA	-0,2231	-0,3118 to -0,1343	<0,0001
	FR vs. ABA	-0,2089	-0,2977 to -0,1201	<0,0001
	EXE vs. ABA	-0,2037	-0,2925 to -0,1150	<0,0001
$pULK1_{S757}$	CTRL vs. ABA	-0,1915	-0,3169 to -0,06618	0,0024
	FR vs. ABA	-0,1432	-0,2685 to -0,01783	0,0225
	EXE vs. ABA	-0,3109	-0,4363 to -0,1856	<0,0001
ULK1	CTRL vs. ABA	-0,3213	-0,5742 to -0,06838	0,0108
	FR vs. ABA	-0,2584	-0,5114 to -0,005499	0,0444
	EXE vs. ABA	-0,4929	-0,7458 to -0,2400	0,0002
$pTFEB_{S211}$	CTRL vs. FR	0,2004	0,06339 to 0,3374	0,0035
	CTRL vs. EXE	0,1655	0,02850 to 0,3025	0,0154
	CTRL vs. ABA	0,1498	0,01280 to 0,2868	0,0297
TFEB	CTRL vs. ABA	0,1862	0,06419 to 0,3082	0,0024
	FR vs. ABA	0,1986	0,07655 to 0,3205	0,0014
	EXE vs. ABA	0,2183	0,09631 to 0,3403	0,0005
Beclin-1	CTRL vs. ABA	-0,1072	-0,2090 to -0,005369	0,0373
	FR vs. ABA	-0,1109	-0,2127 to -0,009066	0,0305
	EXE vs. ABA	-0,1509	-0,2527 to -0,04908	0,0031

Nuclear Fraction		Acute phase		
Tukey's multiple comparisons test		**Mean diff.**	**95% CI of diff.**	**P value**
TFEB	CTRL vs. ABA	-0,3696	-0,5405 to -0,1988	<0,0001
	FR vs. ABA	-0,4177	-0,5886 to -0,2468	<0,0001
	EXE vs. ABA	-0,4554	-0,6263 to -0,2845	<0,0001

Table 4. Detailed mean differences (diff.), 95% Confidence Interval (CI) of difference and p values relative to Tukey's multiple comparisons test following significant interaction of two-way ANOVA analyses of protein levels measured in the dorsal hippocampus of CTRL, FR, EXE and ABA rats at the establishment of the acute phase and presented in figures 1-5 and in supplementary figure 1. Statistical significance was considered when $p < 0.05$, for $\alpha = 0.05$.

**Homogenate**		After recovery		
Tukey's multiple comparisons test		**Mean diff.**	**95% CI of diff.**	**P value**
mTOR	CTRL vs. ABA	-0,08157	-0,1522 to -0,01090	0,0210
	FR vs. ABA	-0,09129	-0,1620 to -0,02062	0,0095
	EXE vs. ABA	-0,09663	-0,1673 to -0,02596	0,0061
LC3-II	CTRL vs. ABA	-0,2753	-0,5459 to -0,004773	0,0454
	FR vs. ABA	-0,2711	-0,5417 to -0,0006020	0,0494
	EXE vs. ABA	-0,3424	-0,6130 to -0,07190	0,0111
Caspase3	CTRL vs. ABA	-0,3230	-0,5589 to -0,08702	0,0063
	FR vs. ABA	-0,3499	-0,5858 to -0,1139	0,0033
	EXE vs. ABA	-0,3250	-0,5609 to -0,08903	0,0060
p62	CTRL vs. ABA	-0,08749	-0,1628 to -0,01218	0,0205
	EXE vs. ABA	-0,1289	-0,2088 to -0,04906	0,0016

**Cytosolic fraction**		After recovery		
Tukey's multiple comparisons test		**Mean diff.**	**95% CI of diff.**	**P value**
$pmTOR_{S2448}$	EXE vs. ABA	-0,05268	-0,08703 to -0,01834	0,0023
$pmTOR_{S2448}$/mTOR	CTRL vs. EXE	0,3853	0,1309 to 0,6396	0,0026
	EXE vs. ABA	-0,3375	-0,5919 to -0,08315	0,0078
Beclin-1	CTRL vs. ABA	-0,03326	-0,06049 to -0,006040	0,0143
	FR vs. ABA	-0,02930	-0,05652 to -0,002075	0,0327
	EXE vs. ABA	-0,03730	-0,06452 to -0,01008	0,0060

**Nuclear Fraction**		After recovery		
Tukey's multiple comparisons test		**Mean diff.**	**95% CI of diff.**	**P value**
TFEB	CTRL vs. ABA	-0,2704	-0,4412 to -0,09961	0,0017
	FR vs. ABA	-0,2112	-0,3821 to -0,04043	0,0131
	EXE vs. ABA	-0,2462	-0,4170 to -0,07534	0,0040

Table 5. Detailed mean differences (diff.), 95% Confidence Interval (CI) of difference and p values relative to Tukey's multiple comparisons test following significant interaction of two-way ANOVA analyses of protein levels measured in the dorsal hippocampus of CTRL, FR, EXE and ABA rats after a seven-day recovery period and presented in figures 1-5. Statistical significance was considered when $p < 0.05$, for $\alpha = 0.05$.

mRNA levels	Acute phase		After recovery	
Normality test : D'Agostino-Pearson omnibus	**Statistics**	**P value**	**Statistics**	**P value**
Becn1	1,714	0,4244	2,627	0,2689
Ctsb	1,801	0,4063	0,2918	0,8642

Table 6. Detailed statistics of D'Agostino-Pearson omnibus normality test related to mRNA levels evaluated in the dorsal hippocampus of CTRL, FR, EXE and ABA rats at the establishment of the acute phase and after a seven-day recovery period and presented in figures 2 and 4. Statistical significance was considered when $p < 0.05$, for $\alpha = 0.05$

mRNA levels	Acute phase		After recovery	
Brown-Forsythe test	**F (DFn, DFd)**	**P value**	**F (DFn, DFd)**	**P value**
Becn1	0,9251 (3, 16)	0,4512	0,9804 (3, 16)	0,4266
Ctsb	1,512 (3, 16)	0,2495	2,898 (3, 16)	0,0674

Table 7. Detailed statistics of Brown-Forsythe test to assess homogeneity of variances related to mRNA levels evaluated in the dorsal hippocampus of CTRL, FR, EXE and ABA rats at the establishment of the acute phase and after a seven-day recovery period and presented in figures 2 and 4. Statistical significance was considered when $p < 0.05$, for $\alpha = 0.05$

mRNA levels		Acute phase		After recovery	
Two-way ANOVA		**F value**	**P value**	**F value**	**P value**
Becn1	Interaction	F (1, 16) = 46,88	P<0,0001	F (1, 16) = 72,97	P<0,0001
	Exercise	F (1, 16) = 2,655	P=0,1228	F (1, 16) = 6,278	P=0,0234
	Food restriction	F (1, 16) = 13,48	P=0,0021	F (1, 16) = 3,089	P=0,0979
Ctsb	Interaction	F (1, 16) = 25,69	P=0,0001	F (1, 16) = 5,059	P=0,0389
	Exercise	F (1, 16) = 4,547	P=0,0488	F (1, 16) = 1,498	P=0,2387
	Food restriction	F (1, 16) = 19,03	P=0,0005	F (1, 16) = 19,14	P=0,0005

Table 8. Detailed F values, degrees of freedom and p values relative to two-way ANOVA analyses of mRNA levels evaluated in the dorsal hippocampus of CTRL, FR, EXE and ABA rats at the establishment of the acute phase and after a seven-day recovery period and presented in figures 2 and 4. Statistical significance was considered when $p < 0.05$, for $\alpha = 0.05$

mRNA levels		Acute phase		
Tukey's multiple comparisons test		**Mean diff.**	**95% CI of diff.**	**P value**
Becn1	CTRL vs. EXE	0,1235	0,02774 to 0,2193	0,0096
	CTRL vs. ABA	-0,1255	-0,2212 to -0,02970	0,0086
	FR vs. ABA	-0,2006	-0,2964 to -0,1049	0,0001
	EXE vs. ABA	-0,2490	-0,3448 to -0,1532	<0,0001
Ctsb	CTRL vs. ABA	-0,5008	-0,8128 to -0,1888	0,0015
	FR vs. ABA	-0,5553	-0,8673 to -0,2432	0,0006
	EXE vs. ABA	-0,7272	-1,039 to -0,4152	<0,0001

mRNA levels		After recovery		
Tukey's multiple comparisons test		**Mean diff.**	**95% CI of diff.**	**P value**
Becn1	CTRL vs. FR	0,2350	0,09485 to 0,3751	0,0010
	CTRL vs. EXE	0,2091	0,06895 to 0,3492	0,0030
	CTRL vs. ABA	-0,1477	-0,2878 to -0,007517	0,0372
	FR vs. ABA	-0,3827	-0,5228 to -0,2425	<0,0001
	EXE vs. ABA	-0,3568	-0,4969 to -0,2166	<0,0001
Ctsb	CTRL vs. ABA	-0,4995	-0,8605 to -0,1385	0,0056
	EXE vs. ABA	-0,5910	-0,9520 to -0,2300	0,0013

Table 9. Detailed mean differences (diff.), 95% Confidence Interval (CI) of difference and p values relative to Tukey's multiple comparisons test following significant interaction of two-way ANOVA analyses of mRNA levels evaluated in the dorsal hippocampus of CTRL, FR, EXE and ABA rats at the establishment of the acute phase and after a seven-day recovery period and presented in figures 2 and 4. Statistical significance was considered when $p < 0.05$, for $\alpha = 0.05$

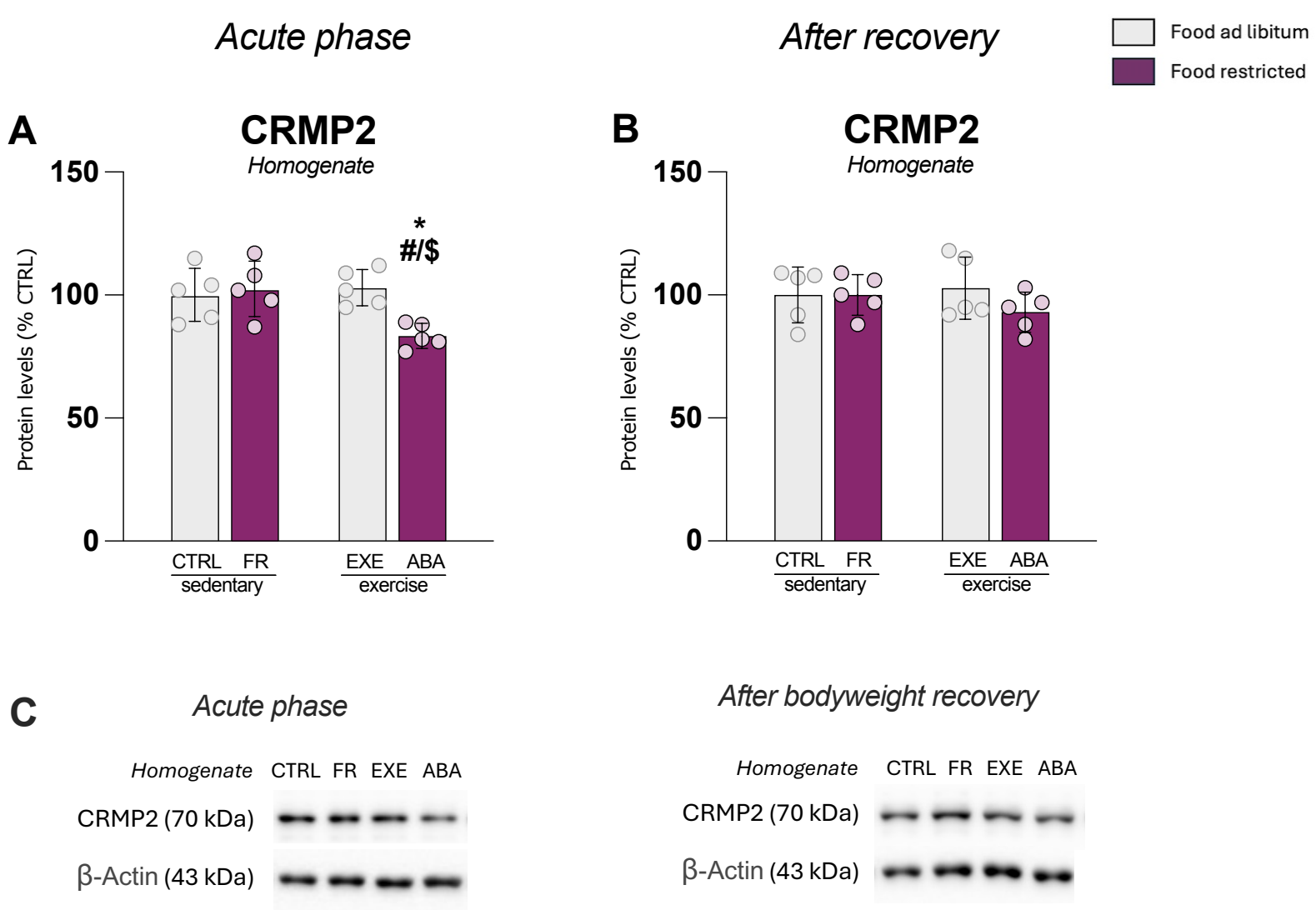

Figure 1. The induction of the ABA phenotype reduces CRMP2 protein levels in the dorsal hippocampus at the acute phase. Panels (**A**) and (**B**) show CRMP2 protein levels in the whole homogenate of CTRL, FR, EXE and ABA rats, at the acute phase and after seven days of bodyweight recovery, respectively. Representative immunoblots for each protein are shown in panel (**C**). Data are expressed as percentage (%) of CTRL and represent the mean ± SD. Two-way ANOVA followed by Tukey's multiple comparison test * $p < 0.05$ vs CTRL; $^{\#}p < 0.05$ vs FR; $^{\$}$ $p < 0.05$ vs EXE. CTRL: control (n=5); FR: food restricted (n=5); EXE: exercise (n=5); ABA: activity-based anorexia (n=5).

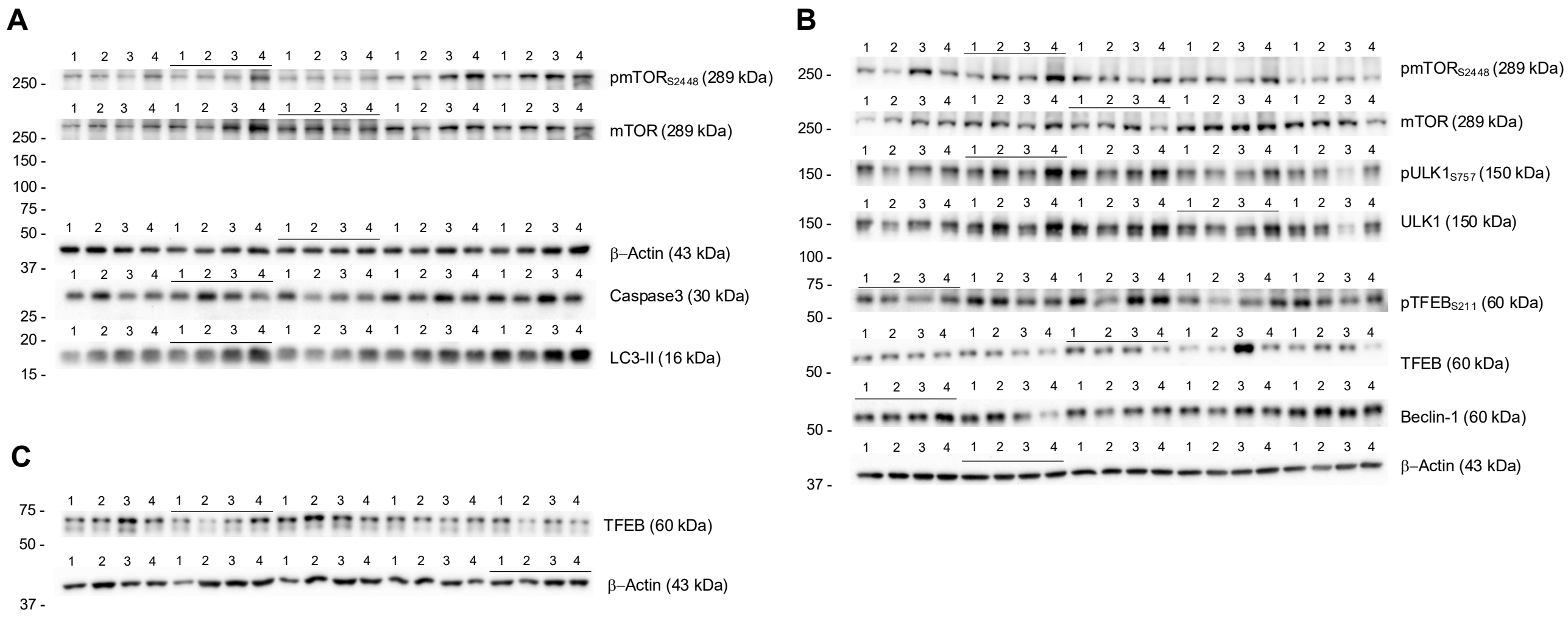

Figure 2. Example of full-size cropped immunoblots related to the protein expression levels of **A)** pmTOR$_{S2448}$ (289 kDa), mTOR (289 kDa), β-actin (43 kDa), Caspase3 (30 kDa), LC3-II (16 kDa) measured in the whole homogenate of the dorsal hippocampus; **B)** pmTOR$_{S2448}$ (289 kDa), mTOR (289 kDa), pULK1$_{S757}$ (150 kDa), ULK1 (150 kDa), pTFEB$_{S211}$ (60 kDa), TFEB (60 kDa), Beclin-1 (60 kDa), β-actin (43 kDa) in the cytosolic fraction of the dorsal hippocampus; **C)** TFEB (60 kDa) and β-actin (43 kDa) in the nuclear fraction of female rats at the acute phase of the anorexic phenotype (PND42), and presented in figures 1,2 and 5. Underlined lanes indicate the representative blots shown in the related figures.

1: CTRL: controls, 2: FR: food restricted, 3: EXE: exercise, 4: ABA: Activity-based anorexia.

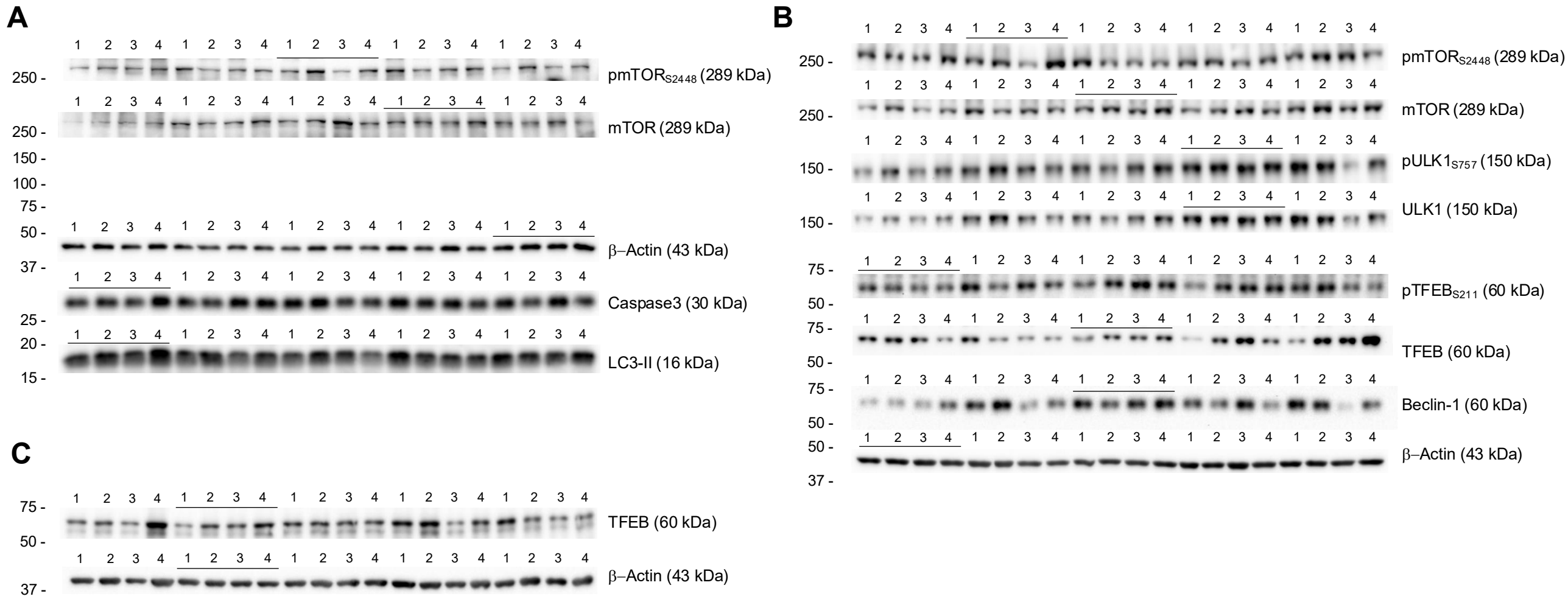

Figure 3. Example of full-size cropped immunoblots related to the protein expression levels of **A)** pmTOR$_{S2448}$ (289 kDa), mTOR (289 kDa), β-actin (43 kDa), Caspase3 (30 kDa), LC3-II (16 kDa) measured in the whole homogenate of the dorsal hippocampus; **B)** pmTOR$_{S2448}$ (289 kDa), mTOR (289 kDa), pULK1$_{S757}$ (150 kDa), ULK1 (150 kDa), pTFEB$_{S211}$ (60 kDa), TFEB (60 kDa), Beclin-1 (60 kDa), β-actin (43 kDa) in the cytosolic fraction of the dorsal hippocampus; **C)** TFEB (60 kDa) and β-actin (43 kDa) in the nuclear fraction of female rats after seven days of bodyweight recovery (PND49), and presented in figures 3-5. Underlined lanes indicate the representative blots shown in the related figures.

1: CTRL: controls, 2: FR: food restricted, 3: EXE: exercise, 4: ABA: Activity-based anorexia.

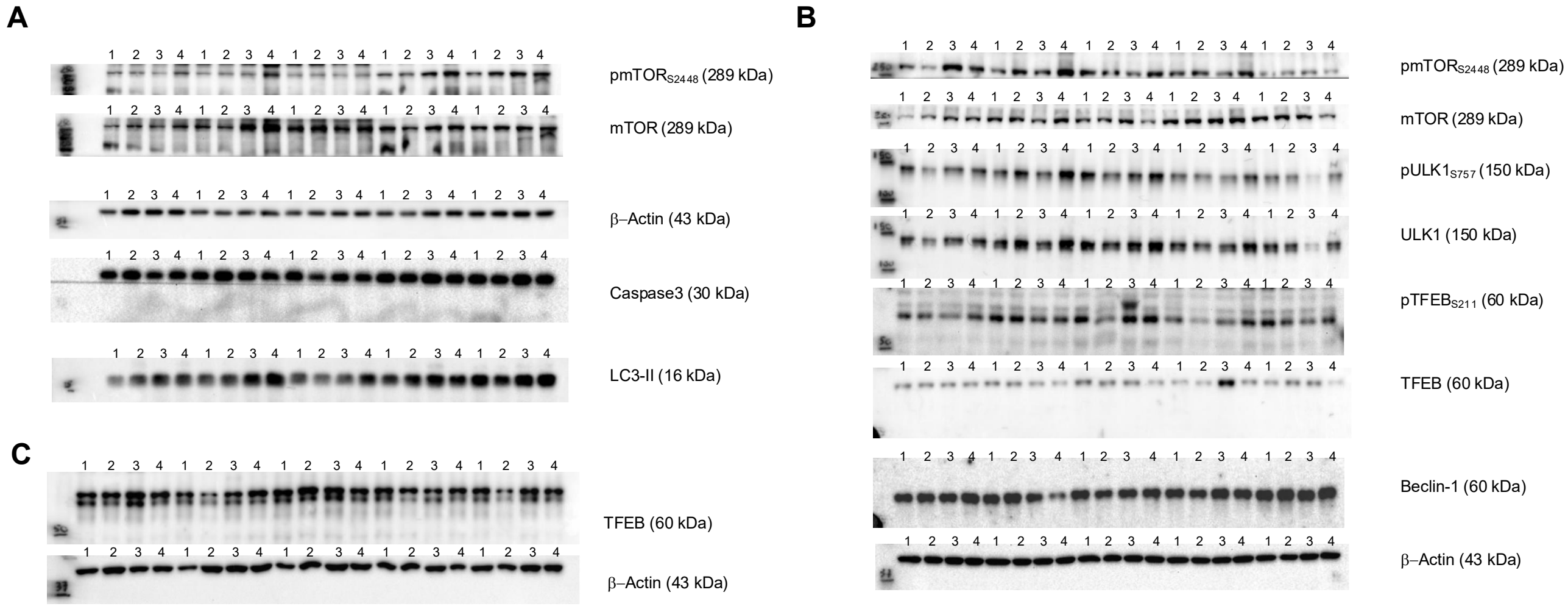

Figure 4. Example of merged images of the membranes showing the labeled protein ladder and the cropped immunoblot related to the protein expression levels of **A)** pmTOR$_{S2448}$ (289 kDa), mTOR (289 kDa), β-actin (43 kDa), Caspase3 (30 kDa), LC3-II (16 kDa) measured in the whole homogenate of the dorsal hippocampus; **B)** pmTOR$_{S2448}$ (289 kDa), mTOR (289 kDa), pULK1$_{S757}$ (150 kDa), ULK1 (150 kDa), pTFEB$_{S211}$ (60 kDa), TFEB (60 kDa), Beclin-1 (60 kDa), β-actin (43 kDa) in the cytosolic fraction of the dorsal hippocampus; **C)** TFEB (60 kDa) and β-actin (43 kDa) in the nuclear fraction of female rats at the acute phase of the anorexic phenotype (PND42), and presented in figures 1,2 and 5.

1: CTRL: controls, 2: FR: food restricted, 3: EXE: exercise, 4: ABA: Activity-based anorexia.

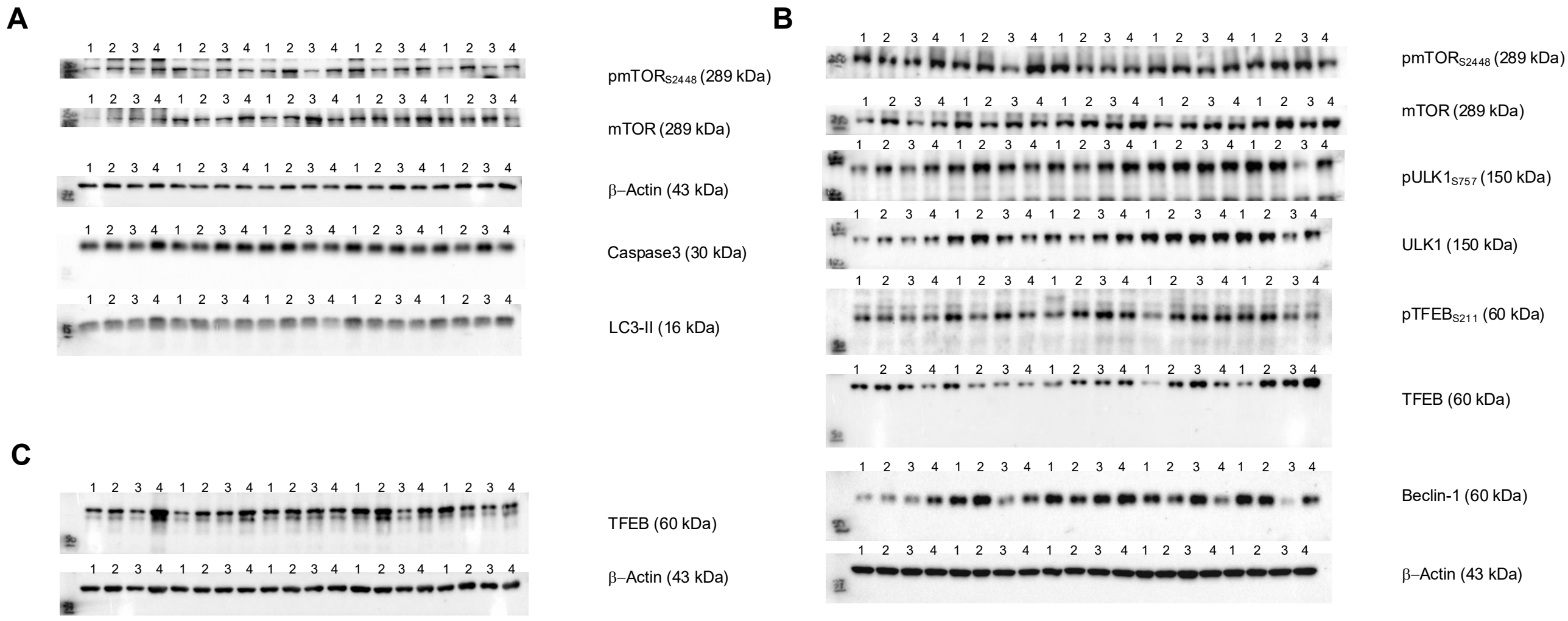

Figure 5. Example of merged images of the membranes showing the labeled protein ladder and the cropped immunoblot related to the protein expression levels of **A)** pmTOR$_{S2448}$ (289 kDa), mTOR (289 kDa), β-actin (43 kDa), Caspase3 (30 kDa), LC3-II (16 kDa) measured in the whole homogenate of the dorsal hippocampus; **B)** pmTOR$_{S2448}$ (289 kDa), mTOR (289 kDa), pULK1$_{S757}$ (150 kDa), ULK1 (150 kDa), pTFEB$_{S211}$ (60 kDa), TFEB (60 kDa), Beclin-1 (60 kDa), β-actin (43 kDa) in the cytosolic fraction of the dorsal hippocampus; **C)** TFEB (60 kDa) and β-actin (43 kDa) in the nuclear fraction of female rats after seven days of bodyweight recovery (PND49), and presented in figures 3-5.

1: CTRL: controls, 2: FR: food restricted, 3: EXE: exercise, 4: ABA: Activity-based anorexia.

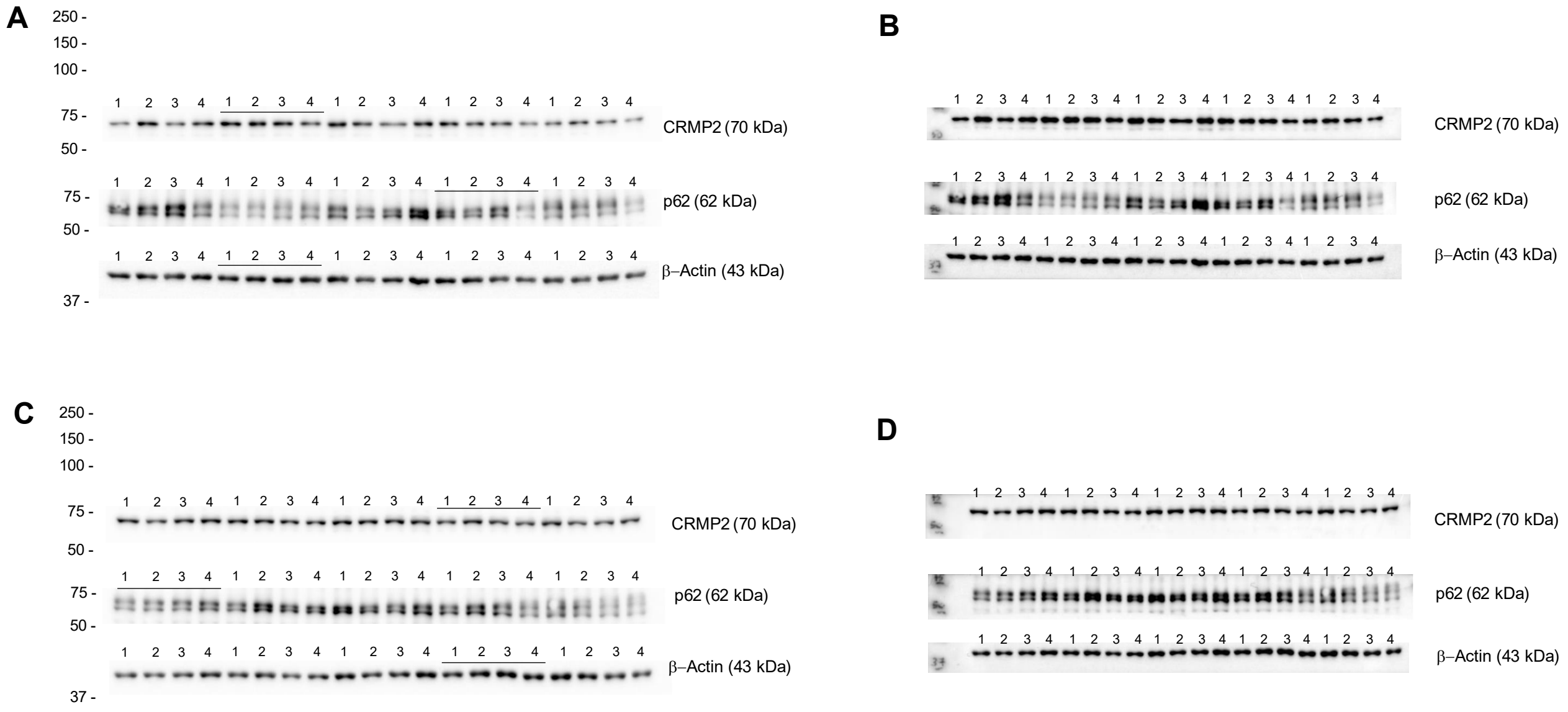

Figure 6. Example of full-size cropped immunoblots and merged images of membranes showing the labeled protein ladder and the cropped immunoblot related to the protein expression levels of **A)**, **B)** CRMP2 (70 kDa), p62 (62 kDa) and β-actin (43 kDa) measured in the whole homogenate of the dorsal hippocampus at the acute phase; **C)**, **D)** CRMP2 (70 kDa), p62 (62 kDa) and β-actin (43 kDa) in the whole homogenate of the dorsal hippocampus after seven days of bodyweight recovery and presented in figures 5 and in supplementary figure 1. Underlined lanes indicate the representative blots shown in the related figures.

1: CTRL: controls, 2: FR: food restricted, 3: EXE: exercise, 4: ABA: Activity-based anorexia.

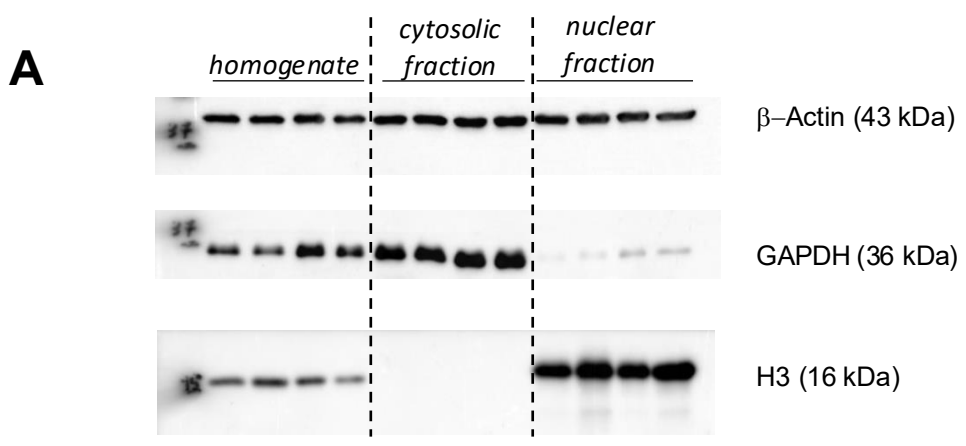

Figure 7. Merged images of the membrane showing labeled protein ladder and the cropped immunoblots related to the protein expression of β-actin (43 kDa), GAPDH (36 kDa) and H3 (16 kDa) measured in the whole homogenate, cytosolic fraction and nuclear fraction of the dorsal hippocampus of CTRL animals, to assess successful subcellular fractionation. CTRL = controls.
